# Study of neurovascular coupling by using mesoscopic and microscopic imaging

**DOI:** 10.1016/j.isci.2021.103176

**Published:** 2021-09-25

**Authors:** Congping Chen, Zhentao She, Peng Tang, Zhongya Qin, Jufang He, Jianan Y. Qu

**Affiliations:** 1 Department of Electronic and Computer Engineering, The Hong Kong University of Science and Technology, Clear Water Bay, Kowloon, Hong Kong, P. R. China; 2 State Key Laboratory of Molecular Neuroscience, The Hong Kong University of Science and Technology, Clear Water Bay, Kowloon, Hong Kong, P. R. China; 3 Center of Systems Biology and Human Health, The Hong Kong University of Science and Technology, Clear Water Bay, Kowloon, Hong Kong, P. R. China; 4 Department of Neuroscience (NS), City University of Hong Kong, Hong Kong, P.R. China; 5 Centre for Regenerative Medicine and Health, Hong Kong Institute of Science & Innovation, Chinese Academy of Sciences, Hong Kong SAR, P.R. China

**Keywords:** Small animal imaging, Optical imaging, Techniques in neuroscience

## Abstract

Neuronal activation is often accompanied by the regulation of cerebral hemodynamics via a process known as neurovascular coupling (NVC) which is essential for proper brain function and has been observed to be disrupted in a variety of neuropathologies. A comprehensive understanding of NVC requires imaging capabilities with high spatiotemporal resolution and a field-of-view that spans different orders of magnitude. Here, we present an approach for concurrent multi-contrast mesoscopic and two-photon microscopic imaging of neurovascular dynamics in the cortices of live mice. We investigated the spatiotemporal correlation between sensory-evoked neuronal and vascular responses in the auditory cortices of living mice using four imaging modalities. Our findings unravel drastic differences in the NVC at the regional and microvascular levels and the distinctive effects of different brain states on NVC. We further investigated the brain-state-dependent changes of NVC in large cortical networks and revealed that anesthesia and sedation caused spatiotemporal disruption of NVC.

## Highlights

• Concurrent mesoscopic and microscopic imaging of neurovascular dynamics
• Spatiotemporal characteristics of neurovascular responses across multiple scales
• Distinct effects of anesthesia and sedation on neurovascular coupling
• Cortex-wide correlation of neuronal activity and cerebral hemodynamics

## Graphical abstract

**Figure:**
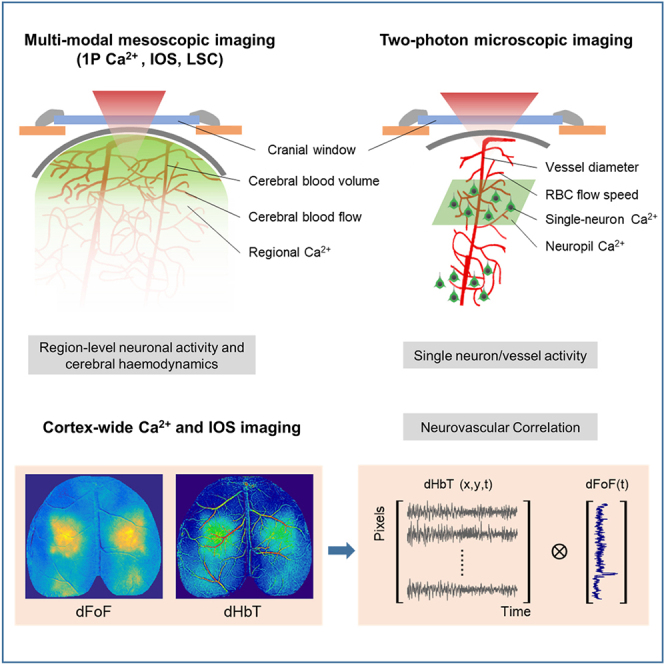

## Introduction

Neuronal activity in the brain occurs in tandem with a complex sequence of vascular and haemodynamic processes that are essential for meeting neuronal energy demands, and oxygen and nutrient requirements (Grutzendler and Nedergaard, 2019; Iadecola, 2017). The regulation of cerebral blood flow (CBF) in response to changes in regional neuronal activity, known as neurovascular coupling (NVC), involves a well-orchestrated interaction of neurons with glial and vascular cells. This interaction transduces specific vasoactive signals into corresponding vascular changes, resulting in CBF increases that are spatiotemporally linked to neuronal activation (Kleinfeld et al., 2011; Zlokovic, 2011). NVC is the biophysical basis for modern functional neuroimaging techniques such as functional magnetic resonance imaging (fMRI) and positron emission tomography (PET), and underlies the roles of vascular dysfunction in normal brain aging(Beishon et al., 2021) and in neurological disorders such as depression(Burrage et al., 2018; Najjar et al., 2013), stroke(Hu et al., 2017; Zoppo, 2010), and Alzheimer's disease(Girouard and Iadecola, 2006; Zlokovic, 2005), etc. However, the cellular and molecular mechanisms that modulate NVC are poorly understood and the extent to which local neuronal and vascular responses are correlated remains unclear (Attwell et al., 2010).

Advances in wide-field imaging techniques have enabled the close monitoring of neuronal activities (via single-photon calcium [1P Ca^2+^ imaging) and cerebral haemodynamics, including cerebral blood volume (CBV) (via intrinsic optical signal [IOS] imaging) and CBF (via laser speckle contrast [LSC] imaging), in the live brains of small animals (Boas and Dunn, 2010; Bouchard et al., 2009; Ma et al., 2016a). However, conventional wide-field imaging techniques have limited spatial resolution and penetration depth, and are thus unable to resolve cellular or microvascular structures. Although two-photon (2P) fluorescence microscopy allows the visualisation of subcellular and capillary details across large depths in the rodent cortex *in vivo* (Helmchen and Denk, 2005; Shih et al., 2012), it has a relatively small field-of-view (<1 mm^2^), which limits its ability to assess neuronal and vascular dynamics over large cortical regions (Ji et al., 2016). The spatiotemporal correspondence between neuronal activities and haemodynamic responses have mostly been studied at either regional (Lake et al., 2020; Winder et al., 2017) or capillary levels (Chow et al., 2020; O'Herron et al., 2016), but not at both. To elucidate the underlying mechanisms of neurovascular signalling, imaging must characterise neuronal and vascular dynamics in live brains at high spatiotemporal resolution and across multiple spatial scales.

In the recent decades, tremendous efforts have been devoted to investigate the possible mechanisms of neurovascular coupling governing the resting-state haemodynamics (Ma et al., 2016a; Winder et al., 2017) and the evoked vascular responses in various sensory cortices, including somatosensory(Gu et al., 2018), visual (Pisauro et al., 2013), olfactory bulb (Vincis et al., 2015) and auditory cortex (Kameyama et al., 2008), and etc. Among which, auditory cortex with a well-known spatially-structured tonal organization, serves as a good model to investigate the connection between localized haemodynamic signals with the evoked neuronal responses at both regional and cellular level, with respect to various auditory processing mechanisms (duration, frequency, and sound pressure level, etc) (Issa et al., 2014; Liu et al., 2019).

In this study, we developed a multimodal optical imaging method that allows both mesoscopic and microscopic *in vivo* imaging of fast neuronal activities and concurrent vascular responses in the mouse cortex. Using a system incorporating four imaging modalities: 1P Ca^2+^, IOS, LSC, and 2P imaging, we investigated the spatiotemporal correlation between sensory-evoked neuronal and vascular responses in the auditory cortex of awake mice. In particular, we studied the effects of anaesthetics and sedatives on neurovascular responses at both the regional and single neuron/vessel levels. Finally, we performed cortex-wide optical imaging to study the NVC in large cortical networks under different brain states.

## Results

### Mesoscopic imaging of sensory-evoked neurovascular dynamics

**Figure 1:**
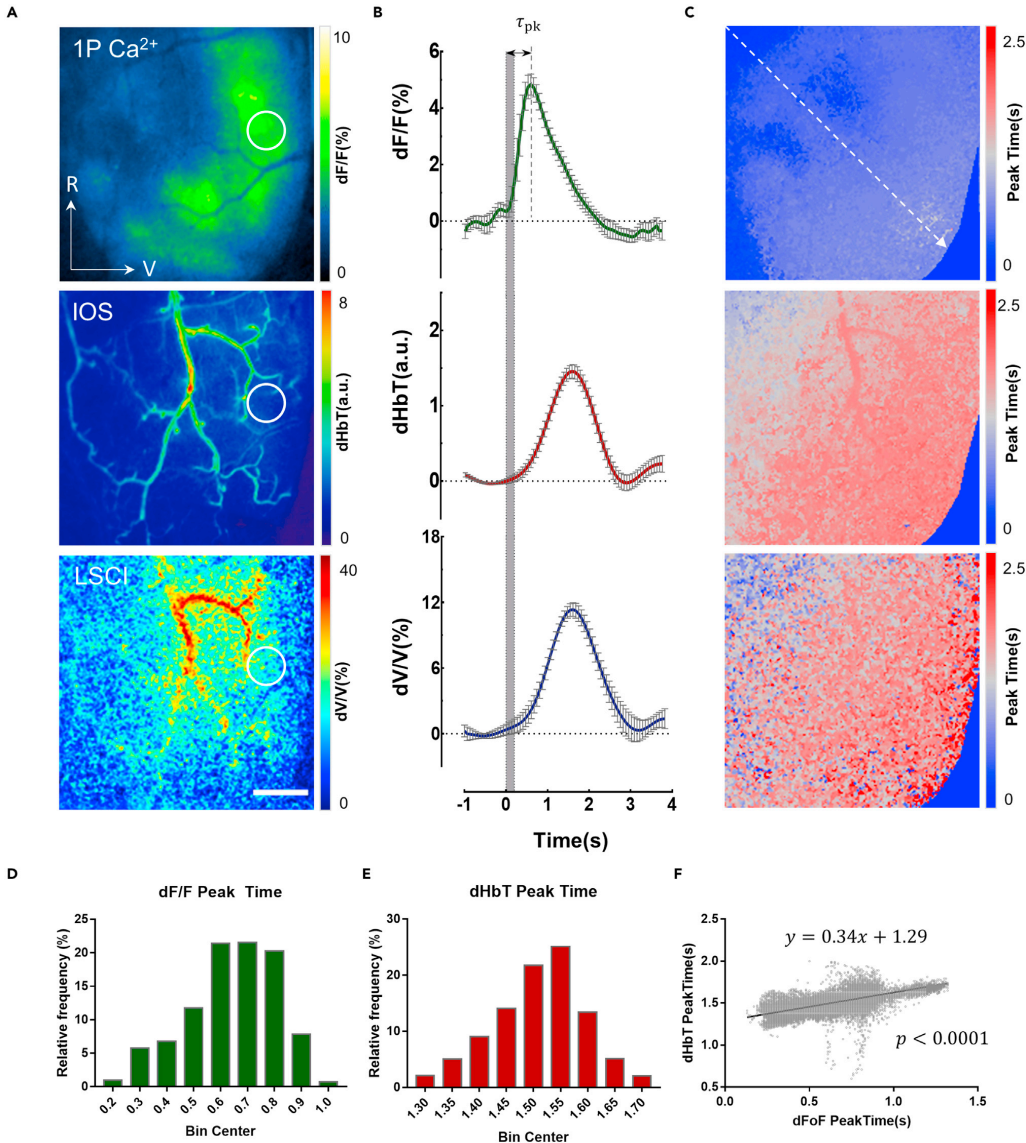
Mesoscopic imaging of sensory-evoked neuronal and vascular responses in the auditory cortex of awake mice (A) Maps of response amplitude to auditory stimulation (80 dBL, 0.2 s) for three different imaging modalities. 1P Ca^2+^: neuronal activity (dF/F); IOS: changes in total hemoglobin (HbT) concentration (dHbT); LSC: relative changes in cerebral blood flow (CBF; dV/V). Anatomical directions: rostral (R); ventral(V); Scale bar: 0.5 mm. (B) Average time courses of dF/F, dHbT and dV/V in responses to auditory stimulation (n = 12) extracted from the parenchymal regions marked by solid circles in (A). The duration of the auditory stimulation (noise, 80 dBL) is shown in grey. Data are presented as means ± standard errors of mean (SEM). (C) Maps of peak time corresponding to the three imaging modalities used in (A), where peak time *τ*_*pk*_ is defined as the time point of maximum amplitude (as shown in (B)). (D and E) Histogram of peak times extracted from the pixels in (C). (F) Scatter plot of dF/F vs. dHbT peak times, with each dot representing a pixel selected along the dashed line in (C).

To visualize neuronal activities and cerebrovascular haemodynamics in the auditory cortex, awake and head-fixed mice were imaged through an open-skull cranial window using a custom-built mesoscopic imaging system (Figure S1). This wide-field optical imaging system integrates three contrast mechanisms: 1P fluorescence, IOS, and LSC (see details in STAR Methods*)* to simultaneously capture the neuronal activities via Ca^2+^ signal (dF/F) and changes in CBV (total haemoglobin concentration or dHbT) and CBF (dV/V) over a relatively large field-of-view (∼10 mm^2^) with high temporal resolution (up to 15 fps). In this work, we used a transgenic mouse line (CaMKII-GCaMP6s) that expressed the genetically encoded Ca^2+^ indicator GCaMP6s in a large population of excitatory neurons across different cortical layers (Figure S2). This transgenic mouse line enabled us to perform functional mapping of the auditory cortex using wide-field Ca^2+^ imaging. Specifically, the tonal map of the auditory cortex was acquired using the Fourier imaging method (Figure S3), during which single tones with ascending frequencies (3–48 kHz) were presented in a periodic manner (Kalatsky et al., 2005). We then used our multi-modal mesoscopic imaging technique to study the sound-evoked neurovascular responses in awake mice. Following white-noise stimulation, we monitored the time course of neuronal activity (dF/F) and the concurrent haemodynamic response (dHbT and dV/V) over the entire auditory cortex (Figure S4). As shown in Figure 1A, the sound-evoked neuronal activity was highly localised to distinct regions within the auditory cortex, whereas IOS imaging showed that blood supply to the active areas was increased, owing to highly dilated surface vessels. Using dual-wavelength IOS imaging, we further observed that the surface vessels that led to a predominant increase in CBV were pial arteries, not veins (Figure S5). Moreover, the elevated CBF observed in these pial arteries matched the spatial pattern of the CBV response, suggesting that CBF is regulated by arteriolar dilation, without concomitant changes in the perfusion pressure, as follows from Poiseuille's law (Rostrup et al., 2005). It should be noted that mesoscopic IOS and LSC imaging provide temporal information of the haemodynamic responses from the entire auditory cortex including both pial vessels and the parenchyma. However, the haemodynamic signals in parenchyma arising from deeper microvasculature are considered to be directly associated with the neuronal activities in the same region (Chen et al., 2011), thus we primarily investigated the neurovascular coupling based on the correlation between haemodynamic signals from parenchyma and the sound-evoked neuronal activation in the auditory cortex. The time-series analysis (Figure 1B) revealed a classical hyperaemic delay between the elevated CBV/CBF levels and neuronal activation (Senarathna et al., 2019). The similar temporal kinetics of CBV and CBF also confirmed their spatiotemporally coupled relationship. To further characterize the spatiotemporal relationship between the neurovascular responses, we computed the peak time maps for the dF/F, dHbT, and dV/V responses, which depicted the average time taken to attain the peak amplitude at each pixel (Figure 1C). Based on these maps, we inferred that neuronal activation, despites its localized amplitude response, showed more dispersed and spatially graded peak-time characteristics (Figure 1D). CBV/CBF also displayed a spatial gradient of peak times in the parenchymal region, with identifiable surface arteries showing relatively longer peak times (Figure 1E). The linear relationship between the peak times of the dF/F and dHbT responses implies that, in the auditory cortex of awake mice, the stimulus-evoked neurovascular responses are spatiotemporally coupled at the mesoscale level (Figure 1F).

### Two-photon microscopic imaging of sensory-evoked neurovascular dynamics

We used multimodal mesoscopic imaging to demonstrate the spatiotemporal correlations of sound-evoked neuronal and vascular responses at the regional level. However, because of the tissue absorption and scattering 1P Ca^2+^ imaging only reflects the depth-weighted summation of neuronal activities that are biased towards the superficial cortex (Ma et al., 2016a; Waters, 2020), and IOS imaging under visible light mainly resolve the changes in haemoglobin changes from cortical vasculature and brain parenchyma to limited depths (<100 μm, Figure S6). Furthermore, LSC imaging relies on the interference of backscattered light, and is also mostly sensitive to the superficial scattering layers (Davis et al., 2016). Therefore, the previous finding revealed by the multimodal mesoscopic imaging mainly arises from the regional responses from the superficial cortex.

**Figure 2:**
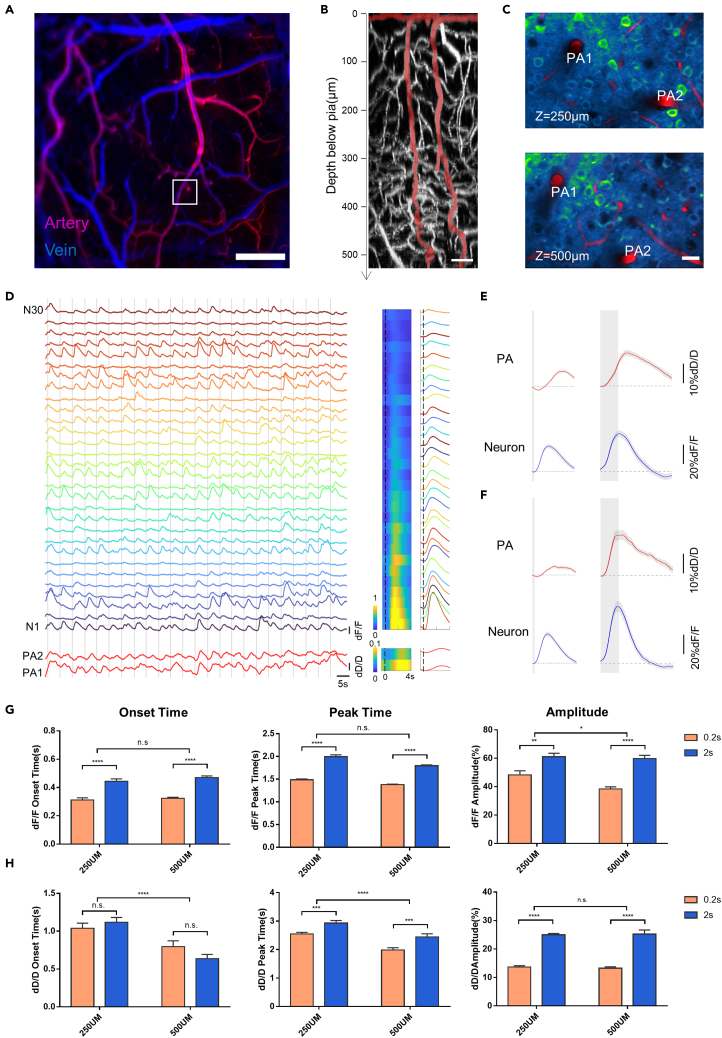
Sensory-evoked responses of auditory neurons and penetrating arterioles in awake mice, acquired with two-photon microscopic imaging (A) Mesoscopic map of surface arteries (red) and veins (blue). Scale bar: 0.5 mm. (B) Maximum intensity projections of two-photon (2P) fluorescence image of Texas Red dextran-labeled vessels in the coronal orientation. Two representative penetrating arterioles (PAs) originating from large surface arteries, highlighted in red. Scale bar: 50 μm. (C) 2P fluorescence images of GCaMP6s-labelled neurons and Texas Red dextran-labeled vessels at two different depths (250 μm and 500 μm) from the primary auditory cortex (white box in (A)). Images are shown as average intensity projections over 600 frames. Scale bar: 20 μm. (D) Left: examples of auditory-evoked Ca^2+^ transients (dF/F) of auditory neurons (N1–N30) and dilation (dD/D) of PAs (PA1 and PA2) at a depth of 250 μm. Right: the corresponding averaged responses of auditory neurons and PAs sorted by amplitude. (E and F) Representative neuronal and PA responses evoked by noise with short- and long-duration stimuli (left: 0.2 s, right: 2 s) at imaging depths of 250 μm (E) and 500 μm (F). Data are presented as means ± SEM. (G and H) Statistics of onset time (left), peak time (middle) and amplitude (right) extracted from the responses of auditory neurons (G) and PAs (H). Onset time is defined as the intercept between the linear fitted line of the rising slope (20–80% of peak amplitude) and the pre-stimulus baseline. Data are presented as means ± SEM. (n = 62 neurons at each depth from two mice; n = 8 PAs at each depth from two mice; ∗: p < 0.0332; ∗∗:p < 0.0021; ∗∗∗:p < 0.0002; ∗∗∗∗:p < 0.0001, two-way ANOVA).

Penetrating arterioles (PAs) that extend from the pial arteries to deep capillary beds, regulating the blood flow through vessel dilation or constriction, are considered to play a pivotal role in functional hyperaemia (Grubb et al., 2020; Nishimura et al., 2007). Previous studies have revealed laminar differences in arteriolar dilation in response to sensory stimulation that supports the retrograde propagation in PAs (Tian et al., 2010), but the correlation between PA dilation and its surrounding neuronal activities remains unclear. To precisely visualize the stimulus-evoked neurovascular responses in deeper cortical regions and at the single neuron/vessel level, we performed 2P microscopic imaging of neuronal activities and vascular dilation in the auditory cortex of awake mice (Figure S7). A fluorescent dye (Texas Red dextran) was retro-orbitally injected into CaMKII-GCaMP6s mice to label the vascular lumen in the cortex. The arterioles were then identified from the artery/vein map pre-obtained via mesoscopic IOS imaging (as shown in Figure 2A). We performed 2P imaging of the PAs and their surrounding GCaMP6s-labeled neurons simultaneously, at two different depths (250 μm apart) along the PAs (Figures 2B and 2C). The diameter changes in the PAs and the Ca^2+^ transients of the surrounding neurons were recorded as the mice were subjected to repetitive auditory stimulations (Figure 2D). The auditory stimulation led to the activation of a large population of excitatory neurons in the superficial and deep cortical layers, although the response amplitudes varied between individual neurons. This sensory-evoked neuronal firing was accompanied by the delayed dilation of PAs, consistent with the previously observed mesoscopic results. The representative neuronal and PA responses to short-duration and long-duration auditory stimulation recorded from two cortical depths are shown in Figures 2E and 2F. We next extracted the onset times, peak times, and amplitudes from the auditory-evoked neuronal and arteriolar responses (Figures 2G and 2H). The auditory-evoked responses of both auditory neurons and PAs were largely dependent on the duration of stimulation. Prolonged stimulation resulted in higher amplitudes and peak times of neuronal and vascular responses. The depth comparisons, however, revealed that the neuronal and vascular responses had different temporal characteristics. Both the onset and peak times of the PAs decreased at deeper cortical regions, as was reported in a previous study (Tian et al., 2010), whereas the surrounding auditory neurons did not show significant laminar differences in terms of onset and peak times, at least not at the same temporal scale as that of vascular responses (0.2–0.5s). Moreover, it has been reported that stimulus-induced neuronal activation emerges earlier in the superficial layer, with the laminar differences in the order of tens of millisecond (Chandrasekaran et al., 2017). These results suggest that the spatial gradients of dilation onset and peak times do not arise from similar laminar gradients of local neuronal signaling, thus providing important insights into the neuronal mechanisms underlying the backpropagation of arteriolar vasodilation (Pfeiffer et al., 2021). The aforementioned results obtained through mesoscopic and microscopic imaging indicate that although neuronal and vascular responses present a linearly-correlated spatial gradient across the cortical surface at the regional level, this linear relationship is not preserved at the single arteriole/neuron level at different cortical depths in awake mice.

### Effect of anesthesia on sensory-evoked neurovascular coupling

**Figure 3:**
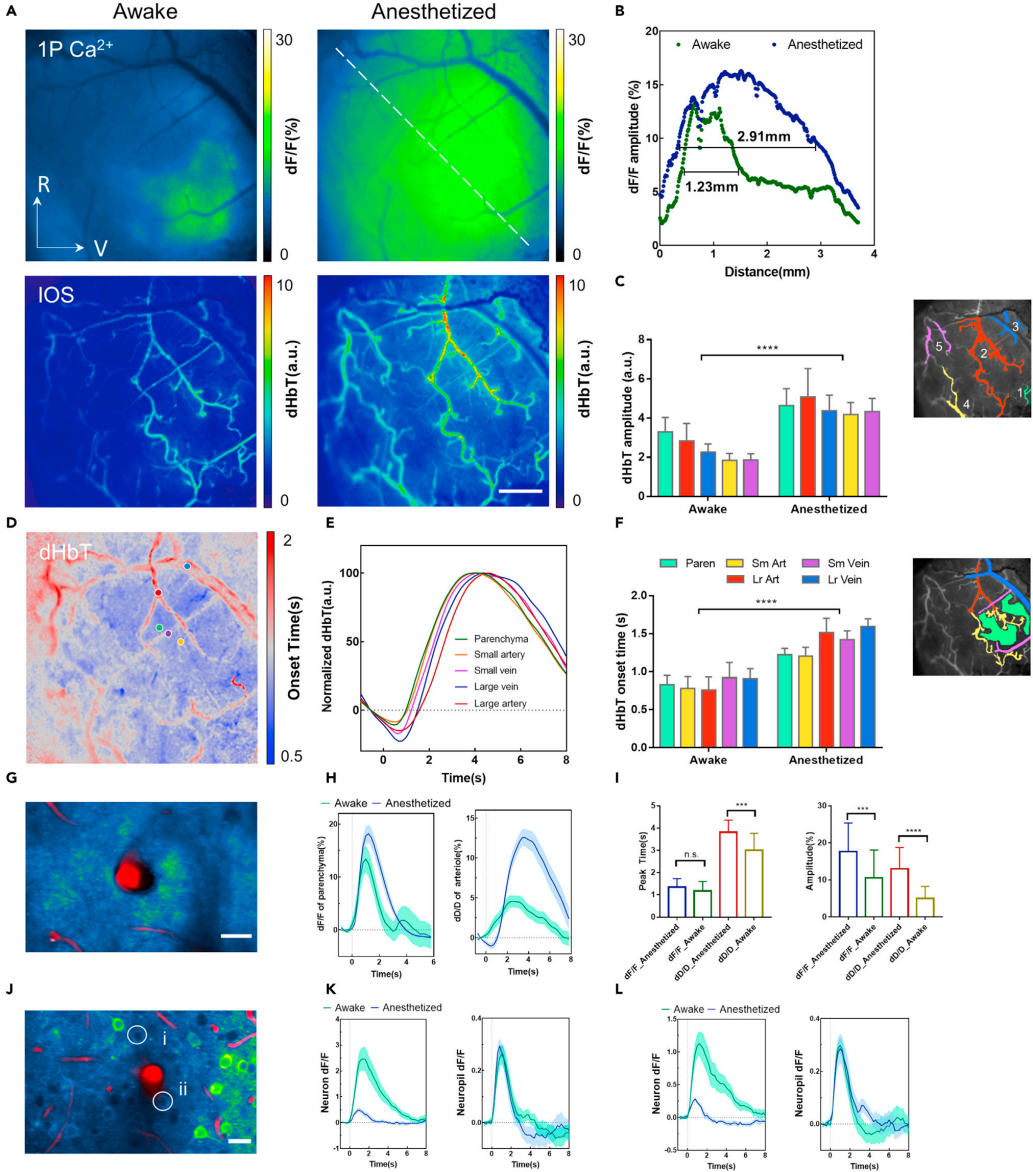
Spatiotemporal relationship of auditory-evoked neurovascular responses in anaesthetized mice (A) Amplitude map of neuronal and vascular responses to auditory stimulation (0.2-s duration) in awake (left) and anaesthetized (right) mice. R: rostral; V: ventral; Scale bar: 0.5 mm. (B) Selected neuronal activity (dF/F amplitude values) along the dashed line shown in (A). Spatial spreads of the neuronal response in terms of full width at half maximum (FWHM). (C) Changes in total haemoglobin (dHbT amplitudes) extracted from the surface arteries as indicated in the colored regions of the inset figure (Cyan: parenchyma; Yellow: small arteries; Purple: small veins; Red: large arteries; Blue: large veins). Data were mean ± standard deviation (SD) (∗∗∗∗p<0.0001, Two-way ANOVA). (D) Map of dHbT onset times in an anaesthetized mouse. (E) Normalized dHbT responses corresponding to surface vessels and parenchyma, as indicated in (D). (F) dHbT onset times extracted from the surface vessels and parenchyma, as indicated in the colored regions of the inset figure; Paren: parenchyma, Sm Art: small artery, Lr Art: large artery, Sm Vein: small vein, Lr Vein: large vein. Data were mean ± SD (∗∗∗∗p < 0.0001, Two-way ANOVA). (G) Two-photon calcium imaging of auditory-evoked (0.2s white noise) neuronal and penetrating arteriole (PA) responses in the superficial cortex (z = 50 μm). The image is shown as an average projection over 600 frames. Scale bar: 20 μm. (H) Neuronal responses (left) and dilation of penetrating arterioles (PAs, right) evoked by 0.2s noise in awake and anaesthetized mice. Data were, means ± SEM. (I) Statistics of peak time (left) and amplitude (right) extracted from the neuronal and arteriolar responses. Data were mean ± SD (∗∗∗p < 0.0002, two-tailed unpaired t test). (J) 2P fluorescence image of neurons and PAs from deeper cortical layers (z = 230 μm). Scale bar: 20μm. (K and L) Exemplar responses of a single neuron (left) its surrounding neuropil (right) evoked by 0.2s noise in awake and anaesthetised mice. (K) and (L) correspond to the numbered neurons (i) and (ii), respectively. Data are presented as means ± SEM.

Cerebral hemodynamics/metabolism and neuronal activities are dependent on factors that influence the physiological brain state, and it is still not known whether NVC occurs reliably in altered brain states (Lecrux and Hamel, 2016; Martin et al., 2006; Winder et al., 2017). In particular, multiple lines of evidence suggest that neuronal activity and vascular hemodynamics are severely impaired by isoflurane-induced global anesthesia, which is routinely used in clinical practice and in the *in vivo* imaging of small rodent brains (Masamoto et al., 2009; Pisauro et al., 2013; Shtoyerman et al., 2000). In this work, we developed a methodology to systematically characterize how the sensory-evoked neuronal and vascular responses are impaired in comparison to the normal awake brain state. First, we used the multimodal mesoscopic imaging to record the neuronal and vascular activities elicited by auditory stimulation from both awake and isoflurane-anaesthetized mice (Figure S8). The amplitude maps of the Ca^2+^ response revealed that the spatial extent of the stimulus-evoked neuronal responses was greatly enlarged in anaesthetized brains, which contrasted with the localized responses observed in awake mice (Figures 3A and 3B). Such enlarged regional responses were also demonstrated by corresponding CBV changes and involved a significant increase in the dilation of additional surface arteries (Figures 3A and 3C), suggesting that the spatial coupling of neurovascular responses is preserved in anaesthetized mice. To further investigate the onset features of these altered neurovascular responses, we computed the maps of onset time for both auditory-evoked responses. The spatiotemporal analysis revealed that the neuronal responses under anesthesia had a gradient of onset time within the expanded responsive field (Figures S9A and S9B). Linear regression analysis of the onset time indicated that the auditory-evoked neuronal responses in anaesthetized mice were initiated in the same cortical region as in awake mice and propagated outwards (Figures S9C and S9D). Mesoscopic IOS imaging enables the characterization of CBV changes from both parenchyma and different vascular components (artery and vein). Analysis of the hemodynamic responses of elevated CBV showed that parenchyma has similar characteristics of sound-evoked responses with that of pial vessels, but pial vessels exhibited a relatively longer onset time than the parenchyma (Figure 3D), which is in keeping with a previous report on the somatosensory cortex of rat models (Chen et al., 2011). The time-series analysis revealed differences in the onset time of the parenchyma and distinct vascular components (Figure 3F). Compared to the awake state, the application of global anesthesia significantly delayed the onset response of the parenchyma and various vascular components at the cortical surface (Figures 3D–3F)). Specifically, dilation onset of large artery (>50μm in diameter) was more delayed relative to that of small artery/arteriole under isoflurane anesthesia (Figure S8). The more delayed dilation in large surface artery may be in part because of the back propagated signaling from deep arterioles. Moreover, isoflurane-induced vasodilation is thought to be vascular structure-dependent, which is more obvious for large arteries than small vessels (Schwinn et al., 1990; Tsurugizawa et al., 2016). Our finding suggests that this vasodilation effect of isoflurane can also lead to the sensory-evoked dilation with delayed onset depending on the vascular structures.

We next used 2P microscopic imaging to measure neuronal Ca^2+^ activities and the dilation of PAs in response to auditory stimulation. As shown in Figures 3G–3I), the surface parenchyma, which primarily consists of apical dendrites and projection axons, showed increased neuronal activity and enhanced arteriolar dilation in anaesthetized mice. Although temporal analysis showed no obvious changes in the onset/peak times of the neuronal responses, the arterioles showed a significantly delayed dilation response (Figures 3G–3I)). These results from the cortical surface were consistent with the aforementioned results obtained through mesoscopic imaging. Further examination of the deeper cortical layers revealed that the Ca^2+^ responses of neuronal somata were notably suppressed by anesthesia, in agreement with the previous report (Issa et al., 2014). However, we found that auditory-evoked responses of the surrounding neuropil were largely unaffected (Figures 3J–3L and S11). These differences in the responses of the neuropil and neuronal somata partly reflect the distinct effects of anesthesia on the local input and output neuronal activities (Kerr et al., 2005).

### Effects of sedation on sensory-evoked neurovascular coupling

**Figure 4:**
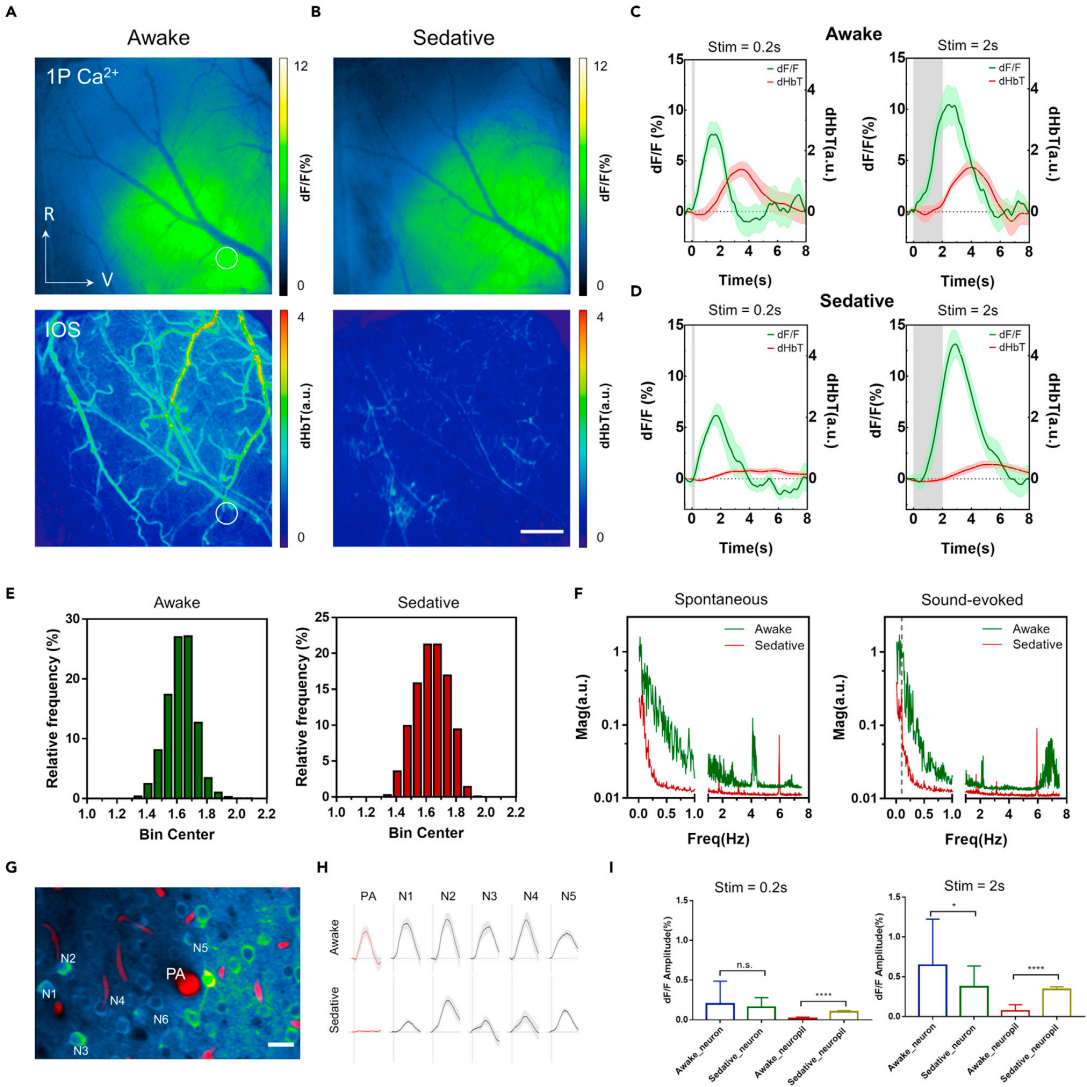
Effects of sedation on neuronal and vascular responses in the auditory cortex (A and B) Amplitude map of neuronal and vascular responses to auditory stimulation (0.2-s white noise) under mouse wakefulness (A) and sedation (B). Scale bar: 0.5 mm. (C and D) Traces of neuronal and vascular responses extracted from the circular region (parenchyma) indicated in (A); Left and right panels correspond to the white noise stimulation of 0.2s and 2s duration, respectively. Data are presented as means ± SEM. (E) Histogram of peak times of dF/F responses in the auditory cortex of the mouse under wakefulness (left) and sedation (right). (F) Magnitude spectra of total hemoglobin changes (dHbT) extracted from surface arteries. Left: spontaneous dynamics without auditory stimulation. Right: sound-evoked activities with 0.2s noise presented at 10s interval (0.1Hz). (G) 2P fluorescence image of neurons and PAs from auditory cortex (z = 240 μm). (H) Representative PA and neuronal responses evoked by 0.2s noise under mice wakefulness and sedation. Data are presented as means ± SEM. (I) Statistics of amplitude extracted from the neuronal and neuropil responses with short- and long-duration stimuli (left: 0.2 s, right: 2 s). Data were mean ± SD (∗p < 0.0332, ∗∗∗p < 0.0002, two-tailed unpaired t test).

Sedation represents yet another brain state of suppressed consciousness and is commonly induced in anaesthetized patients as they undergo clinical surgery. It is also extensively used to reduce the dosage of anesthetics administered during functional brain imaging studies (Becker, 2012). However, there is little evidence to justify the usage of sedative drugs in such studies (Eckley et al., 2020). For instance, chlorprothixene, a sedative widely used in the *in vivo* functional imaging of immobilized rodents (Dana et al., 2014; Drew et al., 2011; Kato et al., 2015), is thought to affect the basal metabolism and vasomotor tone. Despite its popularity, the effects of chlorprothixene on neuronal and hemodynamic activities remain unknown. Therefore, we sought to investigate the effects of sedation on auditory-evoked neurovascular responses by performing mesoscopic and microscopic imaging of the auditory cortices of both awake and sedated mice. Mice were sedated using chlorprothixene administered via intraperitoneal (i.p.) delivery. Mesoscopic imaging showed that sedation had minimal effects on the Ca^2+^ responses of excitatory neurons, in terms of the response amplitude and spatial extent of neuronal activation (Figures 4A and 4B). Moreover, the temporal features of neuronal responses remained stable in awake and in sedated mice (Figure 4E). In terms of vascular responses, the arteriolar dilation elicited by auditory stimulation was severely diminished in sedated mice, although its temporal characteristics did not significantly differ from those of awake mice (Figures 4C and 4D)). Further analysis revealed that the diminished arteriolar responses were partly because of reduced spontaneous dilation (Figure 4F), which may be caused by the strong blocking effect of chlorprothixene on certain receptors (e.g., adrenergic alpha-1) on the vascular smooth muscle cells (Eckley et al., 2020). 2P microscopic imaging showed that the dilation of PAs in sedated mice was also diminished, regardless of the stimulation duration (Figures 4G and 4H)). We also found that auditory-evoked neuronal responses led to substantial decreases (but not the abolition) of responses from neuronal somata in ON- and OFF-neurons (Figure S12). However, sedation globally enhanced the ON-response of the surrounding neuropil to both short-duration and long-duration stimulation (Figure 4I), which resembled the auditory-evoked responses of the ON-neurons in terms of onset/peak time. Overall, our experiments revealed that chlorprothixene-induced sedation will alter the auditory-evoked neuronal and vascular responses by suppressing the dilation responses of arterioles/arteries and enhancing neuropil responses. However, the exact mechanisms are unknown.

### Cortex-wide optical imaging of brain state-dependent neurovascular dynamics

**Figure 5:**
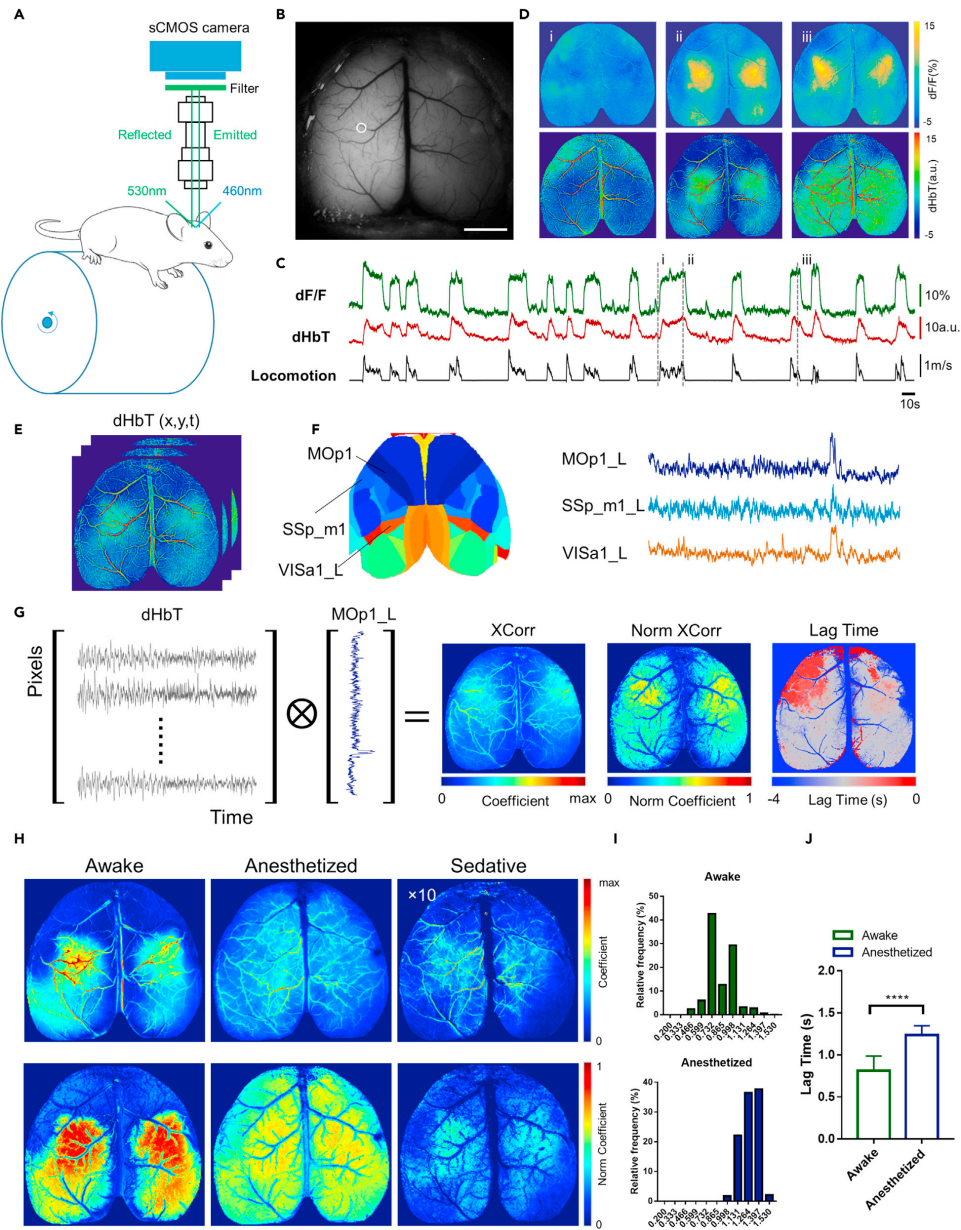
Cortex-wide optical imaging of neuronal and vascular activities and analysis of the neurovascular correlation in different brain states (A) Schematic diagram of the cortex-wide calcium (Ca^2+^) and intrinsic optical signal (IOS) imaging setups. The mouse was head-fixed and placed on a custom-designed rotary treadmill during simultaneous Ca^2+^ and IOS imaging. (B) Reflectance image of the cortex surface through a curved-glass window. Scale bar: 2 mm. (C) Ca^2+^ activities and total hemoglobin changes (dHbT) extracted from the somatosensory cortex (indicated as a white circle in (B)) of an awake behaving mouse. (D) Examples of simultaneously acquired selected neuronal activity (dF/F) and dHbT images from the three time points (i, ii, and iii) indicated in (C). (E–G) Schematic procedure for calculating neurovascular correlation maps (NVCMs). (G) NVCMs of cross correlation (XCorr), normalized cross correlation (Norm XCorr) and lag time are computed between the matrix of dHbT (as shown in (E)) for all pixels over time and neuronal activity dF/F of each temporal component (as shown in (F)). (H) Upper panels: maps of cross-correlation coefficients between neuronal activity (extracted from the primary motor cortex) and the dynamics of changes in total hemoglobin (dHbT); Correlation coefficient under sedative has been increased to 10-fold as indicated for better visualization. Lower panels: the corresponding maps of normalized cross-correlation coefficients. (I) Histogram of lag times (between dHbT and selected neuronal activity (dF/F)) extracted from the primary motor cortex (parenchyma only) in awake (left) and anaesthetized (middle) mice. (J) statistical comparisons of lag times in awake and anaesthetized mice. Data were mean ± SD (∗∗∗∗p < 0.0001, two-tailed unpaired t test).

Thus far, we have revealed the effects of brain states on the mouse auditory cortex using mesoscopic and microscopic optical imaging. To further investigate how different brain states affect NVC in large-scale cortical networks, we performed cortex-wide 1P Ca^2+^ and IOS imaging of CaMKII-GCaMP6s mice implanted with a curved-glass cranial window (Figures 5A and 5B). The mice were head-fixed and placed on a rotary treadmill to facilitate the simultaneous recording of neuronal activities, dHbT dynamics, and real-time locomotion. As shown in Figures 5C and 5D, the neuronal and vascular dynamics in awake behaving mice were highly spatiotemporally correlated. When the mice were made to run fast, neuronal activity, and concurrent CBV elevation were observed in localized regions of the bilateral cortex. To identify different brain regions and extract their neuronal activities, we used a decomposition technique known as localized semi-nonnegative matrix factorization (LocalNMF), which obtains localized spatial components by referencing a standard brain atlas (Oh et al., 2014; Saxena et al., 2020). Cortex-wide optical imaging enables detailed characterization of functional connectivity over the entire dorsal cortex owing to its relatively high spatiotemporal resolution. By comparing the correlation maps based on the Ca^2+^ transients extracted from different cortical regions in awake, anaesthetized and sedated mice (Figure S13), we found that the somatosensory and motor cortices were co-activated and highly correlated in awake mice, whereas other cortical regions showed lower degrees of correlation. This is presumably because mice running behaviour during wakefulness primarily engages the strong neuronal activation in somatosensory and motor cortices. On the other hand, the head-fixed mice remained stationary on the treadmill under anesthesia and sedation. Anesthesia resulted in a markedly higher degree of neuronal correlation between cortical regions, consistent with a previous fMRI-based study of functional connectivity (Paasonen et al., 2018). As for sedation, however, we found that it severely suppressed the correlation between several cortical regions, including the somatosensory and motor cortices.

To evaluate the degree of NVC across the entire dorsal cortex, we computed the correlation maps between the acquired cortex-wide CBV changes (including parenchyma and pial vessels) and the neuronal activities extracted from individual cortical regions (Figures 5E–5G)). Only the correlations in the regions without pial vessels were used for analysis of neurovascular coupling dynamics. The neurovascular correlation maps (NVCMs) illustrate how cortex-wide CBV changes are spatiotemporally related to the neuronal dynamics of a single cortical region. The NVCMs for distinct cortical regions differed significantly, indicating that the neuronal activities of various cortical regions contribute to arteriolar dilation and the resulting CBV elevation to varying extents (Figure S14). We then computed the NVCMs obtained under different brain states (Figure 5H). In awake behaving mice, the NVCMs of the primary motor cortex matched the co-activation of the somatosensory and motor cortices with a high level of cross-correlation (Pearson's correlation r = 0.701 ± 0.19, mean ± standard deviation [SD]) in localized regions. In anaesthetized mice, the NVCMs showed a widespread correlation pattern that involved nearly all surface arterioles and a significantly decreased neurovascular correlation (r = 0.539 ± 0.12). A lag-time analysis revealed that functional hyperemia in anaesthetized mice was substantially delayed compared to that in awake mice (Figures 5I and 5J)). In sedated mice, neurovascular correlation was totally disrupted, primarily because of abolished arteriolar dilation. These observations are consistent with the aforementioned results of mesoscopic imaging, and thus provide key insights into how NVC in large-scale cortical networks is affected by different brain states.

## Discussion

Previous studies based on a variety of *in vivo* imaging tools have greatly facilitated the understanding of the coupling between neuronal activity and concurrent cerebral hemodynamics. However, debate continues on the cellular and molecular mechanisms that underlie such neurovascular signalling and the extent to which the principle of NVC holds. This partly reflects the limitations of existing imaging techniques, which provide information at the regional scale that lacks microvascular and cellular resolution or information at the microvascular scale that has a limited field-of-view. In this study, we developed a multi-scale imaging method that combines mesoscopic and microscopic optical imaging to systematically investigate neuronal activities and vascular hemodynamics at the single neuron/vessel and cortex-wide levels. Specifically, we first investigated sensory-evoked neuronal and vascular responses in the auditory cortex of awake mice using this combined mesoscopic and microscopic imaging technique. We then examined the effects of different brain-state effects (anesthesia and sedation) on NVC in the auditory cortex, and further extended this to cortex-wide analysis.

Our study demonstrated the distinctive relationship of NVC in different brain states. In awake mice, mesoscopic imaging revealed that the auditory-evoked responses of neurons and cerebral vessels showed a linearly-correlated spatial gradient of peak time across the cortical surface, but the 2P microscopic imaging revealed that this linear relationship was not preserved at the single arteriole/neuron level at different cortical depths.

Under isoflurane anesthesia, the spatial extent of the neuronal response was enlarged at the regional level, and arterioles/arteries also show delayed and enhanced dilation responses. At the single-neuron level, the responses of the neuronal somata were suppressed by anesthesia, whereas the sound-evoked responses of the surrounding neuropil were barely affected. It should be noted the isoflurane effect on the evoked dilation of arteries/arterioles may be dose-dependent. Although the low-dose (1% in our study) isoflurane enhances the evoked vasodilation in auditory cortex, consistent with a previous study in somatosensory cortex based on forepaw stimulation (Masamoto et al., 2009), isoflurane of high doses (>1.5%) will lead to decreased vascular responses (Knutsen et al., 2016; Tsurugizawa et al., 2016). This may be explained by the fact that isoflurane itself is a vasodilator and it increases systemic vasodilation at higher dose (Conzen et al., 1992; Schwinn et al., 1990). The low-dose (1%) isoflurane we used only resulted in minimal systemic dilation, thus vasodilation response can occur upon sensory stimulation. However, a high-dose isoflurane can lead to drastic systemic vasodilation (Turner et al., 2020) and thus impair the further evoked dilation.

In mice sedated with chlorprothixene, arteriolar dilation was significantly suppressed and resulted in minor elevation of the CBV in response to auditory stimulation. Auditory-evoked neuronal responses, however, were barely affected by sedation at the regional level. At the single-neuron level, the auditory neurons showed decreased responses to both short and prolonged stimulation, whereas the surrounding neuropil showed enhanced auditory-evoked responses.

The effects of different brain states on NVC were in agreement with the observations from cortex-wide imaging. Anaesthesia led to a widespread decrease in the correlation between neuronal activity and CBV elevation, whereas sedation resulted in disruption of neurovascular correlation in large-scale cortical networks. Collectively, these mesoscopic and microscopic imaging results suggest that neurovascular relationships can differ widely at regional and single neuron/vessel levels, and observation combining both spatial scales is crucial for the comprehensive understanding of the nature of NVC. It is also evident that neuronal and vascular responses are largely dependent on brain states, which provides insights into the molecular signaling mechanisms that govern NVC.

Furthermore, our multi-scale imaging approach may also be valuable to investigate how well the vascular hemodynamics can be predicted by the evoked neuronal activity at different spatial scales (Ma et al., 2016a), in a way to facilitate the interpretation of data in modern neuroimaging techniques. The multi-scale imaging approach holds great potential to investigate the nature of neurovascular coupling as well as neurovascular dysfunction under various pathological conditions such as Alzheimer's disease, hypertension, stroke, depression/stress, and etc., and facilitate pharmacological and therapeutic studies of drugs with actions on the related brain disorders.

## Limitations of the study

It should be noted that although our study only revealed the brain-state effect on excitatory neurons and their correlation with the vascular responses, interneurons also play critical roles in neurovascular coupling (Echagarruga et al., 2020; Uhlirova et al., 2016). In addition to arteriolar dilation for blood flow regulation, capillary is also considered to play active roles in modulation of blood flow mediated through the contractile pericytes (Hall et al., 2014; Hartmann et al., 2021) or the deformable red blood cells (Wei et al., 2016), which challenges the conventional hypothesis of passive capillary perfusion. We further demonstrated that 2P microscopic imaging can be used to simultaneously measure the arteriolar dilation and blood flow/dilation in downstream capillaries during auditory-evoked neuronal activation, thus to extend the study of neurovascular coupling to capillary level. In our study, we found no obvious changes in blood flow or dilation for the measured capillaries (n > 40), except one outliner with increased blood flow (Figure S15), presumably because it may be a first-order capillary or micro-arteriole. However, it must be confirmed with a large-scale study in the future. On the other hand, the possible reason for no observed capillary dilation may be due to the limited resolution (>0.3μm) of 2P microscopy relative to the small changes (Δ < 0.15μm) of capillary diameter (Grutzendler and Nedergaard, 2019).

## STAR★Methods

### Key resources table

**Table:** 

REAGENT or RESOURCE	SOURCE	IDENTIFIER
**Antibodies**		

Rabbit anti-NeuN	Abcam, UK	Cat# ab177487;RRID:AB_2532109
Donkey anti-rabbit 405	Jackson Immunoresearch, US	Cat# 711-475-152;RRID:AB_2340616

**Chemicals, peptides, and recombinant proteins**		

Texas Red dextran	Thermo Fisher, US	Cat No. D1830
Evans Blue	Sigma-Aldrich	Cat No. E2129
Isoflurane	AlfaMedic, HK	Cat No. ISOVET250
Chlorprothixene	Tokyo Chemical Industry Co.	Cat No. C3505

**Experimental models: organisms/strains**		

Mouse: CaMKII-Cre	Jackson Laboratory, US	Stock No: 005359
Mouse: Ai162	Jackson Laboratory, US	Stock No: 031562

**Software and algorithms**		

ImageJ	NIH, US	ImageJ 1.52
MATLAB	MathWorks	MATLAB 2019a
LocalNMF	https://github.com/ikinsella/locaNMF	NA

### Resource availability

#### Lead contact

Further information and requests for resources and reagents should be directed to and will be fulfilled by the Lead Contact, Jianan Y. Qu (eequ@ust.hk).

#### Materials availability

This study did not generate new unique reagents.

### Experimental model and subject details

The double transgenic mouse line, CaMKII-GCaMP6s, was obtained by crossbreeding CaMKII-Cre (Tg[Camk2a-cre] T29-1Stl, Stock No: 005359, Jackson Laboratory, US) homozygous male mice with Ai162 (Ai162[TIT2L-GC6s-ICL-tTA2]-D, Stock No: 031562, Jackson Laboratory) heterozygous female mice. The mice were housed in the Animal Facility of the City University of Hong Kong. In this study, we used young adult mice with age between 8 and 12 weeks, in which the hearing loss issue is negligible (Ison et al., 2007). All animal procedures were conducted in accordance with the guidelines of the Animal Care Facility of the Hong Kong University of Science and Technology (HKUST) and were approved by the Animal Ethics Committee at HKUST.

### Method details

#### Open-skull window in the auditory cortex

To prevent brain swelling and inflammation, the mouse was treated with dexamethasone (0.2 μg/mg) solution 1 h prior to surgery. After anaesthetisation with pentobarbital sodium (50 mg/kg), the mouse was secured on a head-fixing device (SG-4N, Narishige, Japan) for the cranial window implantation. Briefly, after the section of skull covering the auditory cortex was exposed and cleaned with ethanol, a high-speed drill (Strong 204, SAESHIN, Korea) equipped with a 0.5-mm steel burr (19007-05, Fine Science Tools, US) was used to perform a craniotomy (3 × 3 mm^2^) over the auditory cortex with a centre at 3mm posterior to the bregma point and 1.5mm lateral to the rhinal fissure, whilst leaving the dura intact. Any bleeding was stopped using a compressed sponge soaked in saline. A sterile coverslip (3 × 3 mm^2^, 0.2-mm thick) was gently placed over the exposed dura and stabilised onto the skull by applying a small amount of cyanoacrylate adhesive on the edge of the coverslip. A thin layer of adhesive cement (C&B Metabond, Parkell, US) was then applied to the skull and allowed to dry and harden, after which a small amount of dental acrylic was used to cover the skull and the coverslip rim. A rectangular head-plate with a circular hole (8-mm diameter) in its centre was then attached to the cranial window, to allow for head mounting during *in vivo* imaging. After this surgical preparation, the mouse was allowed to recover from anaesthesia and then returned to its home cage. The imaging experiments were conducted after a recovery period of at least 2 weeks.

#### Curved glass window for cortex-wide imaging

A curved glass with a trapezoidal shape (9 × 8 mm^2^, 0.2-mm thick, 10-mm radius curvature) was made by heating and pressing against a cylindrical graphite mold (Kim et al., 2016). The curved glass was cleaned with 75% ethanol before surgical implantation. Briefly, the mouse was administered dexamethasone (0.2 μg/mg) and anaesthetised with pentobarbital sodium (50 mg/kg) 1 h prior to surgery. The mouse skull was exposed by removing the scalp and the periosteum, and then a craniotomy that matched the outline of the curved glass was performed using a high-speed drill. After the groove was gradually deepened by drilling, the central piece of bone was disconnected from the surrounding skull by gently elevating its edges with a pair of forceps. Any bleeding was stopped using a compressed sponge and the curved glass was gently positioned onto the exposed, intact dura. A small amount of cyanoacrylate adhesive was used to glue the glass to the surrounding skull, and then a thin layer of dental acrylic was applied to both the skull and the edge of the glass to stabilise the window. Finally, a custom-designed head plate with a curvature (12-mm radius) matching that of the mouse skull was attached to the skull with dental acrylic, for head-fixation during *in vivo* imaging experiments. The experiments were conducted after a recovery period of at least 2 weeks.

#### Multimodal mesoscopic imaging

Mesoscale imaging of neuronal activity and vascular dynamics in the auditory cortex was conducted in mice that were head-fixed within a custom-built upright multimodal optical-imaging system (Figure S1). This system is capable of multi-contrast imaging, namely 1P Ca^2+^, IOS and LSC imaging.

##### 1P Ca^2+^ imaging

A high-brightness blue light-emitting diode (LED) with a central wavelength of 460 nm was used for GCaMP6s excitation. After passing a collection lens and a band-pass filter (460 nm/60 nm, Chroma, US), the fluorescence was directed into a 4× dry objective (UPlanApo, 0.16 NA, 13 mm W.D., Olympus, Japan) via a dichroic mirror (T470lpxr, Chroma). The GCaMP6s fluorescence image was then captured by a 16-bit scientific complementary metal-oxide-semiconductor (sCMOS) camera (Panda 4.2, PCO, German) via a relay lens consisting of the objective and a tube lens, yielding a ∼3.4 × 3.4 mm^2^ field-of-view with up to 2048 × 2048 pixels/frame. The excitation power projected onto the cortex was adjusted to approximately 10 mW and images were acquired at 30 frames/second (fps) with 2 × 2 spatial binning.

##### IOS imaging

The light source was consisted of 12 LEDs mounted around the objective in a circular configuration using a 3D-printed component (insert in Figure S1). Two types of LEDs (six of each type) with alternating colours (green: 530 nm; blue: 460 nm) were evenly spaced, to provide homogeneous illumination of the sample. Green LEDs were chosen as they fall at the isobestic point of deoxyhaemoglobin/oxyhaemoglobin (HbR/HbO_2_) absorption and could be used to measure the changes in total haemoglobin concentration $\left(\Delta HbT=\Delta HbR+\Delta HbO_{2}\right).$ The oximetric changes in HbT were obtained using both green and blue LEDs. The green and blue LEDs were controlled by a microcontroller board (Arduino UNO, US) that received a transistor-transistor logic (TTL) signal from the sCMOS camera that indicated the time window of light exposure. To perform dual-wavelength IOS imaging, the 1P module was removed. The green and blue LEDs were modulated by two down-sampled TTL pulse trains (with a phase difference of π) that were synchronised with the TTL signal of the sCMOS camera. The excitation powers of the green and blue LEDs were separately tuned by the LED driver to approach a near-saturation level for the camera, which was run at 30 fps with 2 × 2 spatial binning. To conduct simultaneous 1P Ca^2+^ and IOS imaging (HbT only), the blue LED ring lights were replaced with a single blue LED for 1P excitation.

##### LSC imaging

The speckle was generated by shining a collimated 635-nm laser (CPS635R laser module, Thorlabs, US) beam directly onto the cranial window. The laser light was separated from the green light using a second dichroic mirror (T560lpxr, Chroma). The image was captured by another sCMOS camera (Panda 4.2, PCO) that shared the same focal plane with the camera used for 1P Ca^2+^/IOS imaging. These images were acquired at 1024 × 1024 pixels/frame with a temporal resolution of 30 fps.

#### 2P microscopic imaging

2P microscopic imaging was performed using a custom-built upright microscope (Figure S5), based on a previously used imaging setup (Chen et al., 2018). Briefly, the excitation light was delivered by a tuneable mode-locked titanium:sapphire laser (Chameleon Ultra II, Coherent, US). After collimation by a variable beam expander, the laser beam was directed to X-Y galvanometric scan mirrors (6210H, Cambridge Technology, US). The intermediate plane of the Galvo-X and Galvo-Y mirrors was conjugated to the back focal plane of a water-immersion objective (XLUMPLFLN20XW, 1.0 NA, 2 mm W.D., Olympus) via a scan (LSM03-BB, Thorlabs) and a tube lens (TTL200MP, Thorlabs). The fluorescence collected by the objective was separated from the excitation light by a dichroic mirror (705LP, Chroma). Green and red fluorescence were then separated using another dichroic mirror (575LP, Chroma) and directed to two current photomultiplier (PMT) modules (H11461-01 and H11461-03, Hamamatsu, Japan). Two band-pass filters (FF03-525/50-25, Semrock, US and 620 nm/60 nm, Chroma) placed before the PMTs were used to select the fluorescence wavelengths for detection. The microscope was controlled and images were acquired using a multifunction data-acquisition (DAQ) device (PCIe-6361, National Instruments, US) running a custom-written C# program.

For simultaneous 2P imaging of neuronal activity and vascular dynamics, a fluorescent dye Texas Red dextran (70 kDa, Cat No. D1830, Thermo Fisher, US) was retro-orbitally injected (0.25 mg/kg) to the CaMKII-GCaMP6s mice 1 h prior to the imaging experiment to label the blood plasma in the vascular lumen. The mice were placed on a head-mounting stage with angle adjusters (MAG-2, Narishige) and their bodies were restricted within a plastic conical tube to minimise motion during 2P imaging. The laser is tuned at 920 nm for excitation of both GCaMP6s and Texas Red, with the post-objective power adjusted between 15 and 60 mW for different imaging depths (0–550 μm below pia). Neuronal activity and PA dilation were recorded via full frame (256 × 166 pixels) imaging at a frame rate of 5 Hz. The velocity of red blood cells (RBCs) in the capillaries and dilation of horizontal vessels were measured using line-scan imaging at a line acquisition rate that ranged between 0.5 kHz and 1 kHz.

#### Cortex-wide optical imaging

The animals were head-fixed on a custom-designed rotary treadmill apparatus placed under a custom-built optical macroscope for cortex-wide Ca^2+^ and IOS imaging. A high-power blue light source (450-nm LED) was used to excite the GCaMP6s to generate fluorescence, which was collected by a pair of video lenses that yielded a total magnification of ∼1.3× and passed through a band-pass filter (FF03-525/50-25, Semrock). An array of green LEDs (four LEDs with a central wavelength of 530 nm) were used to project homogeneous illumination onto the cortex for IOS imaging. GCaMP6s fluorescence and green-light reflectance images were captured by a scientific camera (sCMOS, Panda 4.2, PCO) in an interleaved manner at a frame rate of 30 Hz and frame size of 512 × 512 pixels. The TTL exposure signal from the camera output was used to sequentially control and synchronise the illumination of the blue and green LEDs. The treadmill apparatus was equipped with a rotary encoder (E40H6-2000-3-V-5, OMRON, Japan) to record the location/speed of behaving mice during cortex-wide optical imaging.

#### Auditory stimulation

The imaging setup for integrating the mesoscopic and microscopic systems was placed in a custom-designed sound-attenuated chamber. The background noise inside the chamber was negligible (<40 dBL), and consisted mainly of low-frequency components (<1 kHz). For sound calibration, an acoustic transducer (Type 4191-B-001, Brüel and Kjær, Denmark) controlled by a conditioning amplifier (Type 2690, Brüel and Kjær) was placed 10 cm from a free-field speaker (MF1, Tucker-Davis Technologies, US) controlled by a synthesised function generator (DS345, Stanford Research Systems, US). The analogue output from the conditioning amplifier was fed into a DAQ board (PCIe-6361, National Instruments) using a custom-written MATLAB (MathWorks, US) program. The sound-pressure level was calculated after filtering out the low-frequency noise and high-frequency harmonics. Sounds were delivered by the free-field speaker placed 10 cm from left ear of the mouse. The sound stimuli consisted of a single tone (3–48 kHz, 85 dBL) with sinusoidal amplitude modulation (SAM; 20-Hz modulation frequency) and white noise (80 dBL).

Fourier imaging (Kalatsky, 2003) was used for tonal mapping of the auditory cortex, with periodic stimulation of SAM tones. Briefly, the stimulation pattern consisted of five SAM tones (3, 6, 12, 24 and 48 kHz) that lasted for 0.2 s each and were presented at 3-s intervals between consecutive frequencies. The SAM tone series of ascending frequencies were repeated 16–32 times during mesoscopic optical imaging.

To investigate the sound-evoked responses of neurons and vessels, the auditory stimuli were delivered as white noise (80 dBL). Stimuli of short (0.2 s) or long (2 s) duration were presented repeatedly at intervals of either 5 s or 10 s, respectively, during a single imaging trial that typically contained 12–24 repetitions of the auditory stimuli. Typically, 2–3 trials were conducted for each auditory stimulus. To avoid mouse adaptation to the passive sound stimulation, repetitive stimuli were presented with less than 50 trials in total for each mouse.

In this study, all auditory stimulation used to elicit the evoked neuronal and vascular responses refer to the white noise (80 dBL) of 0.2s duration, unless otherwise mentioned.

#### Immunohistochemistry

After completing the *in vivo* imaging experiments, the animals were deeply anaesthetised with pentobarbital sodium (200 mg/kg, i.p.) and then sacrificed by transcardially perfusion with 30 mL 1X phosphate buffered saline (PBS), followed by 30 mL 4% paraformaldehyde (PFA). After overnight immersion in PFA maintained at 4°C, the mouse brains were coronally sliced into 50-μm-thick sections using a vibratome (VT1000 S, Leica, US). The brain slices were then rinsed four times (7 min each) in PBS and incubated in blocking solution (5% normal goat serum and 0.3% Triton X-100 in 1X PBS) for 1.5 h at room temperature. Immunostaining was performed using a primary antibody against rabbit anti-NeuN (1:2000, ab177487, Abcam, UK) in a blocking solution for 24 h, maintained at 4°C. The slices were then subjected to four washes in PBS, each lasting 15 min, following which they were incubated with a secondary antibody (donkey anti-rabbit 405 [1:500, 711-475-152, Jackson Immunoresearch, US]) with 0.1% Triton X-100 in 1X PBS (PBST) for 2 h at room temperature. The slices were finally rinsed four times in 1X PBS (7 min for each rinse). The brain slices were then mounted on a glass slide with 70% glycerol in 1X PBS and fluorescence images were acquired using an epi-fluorescence microscope (Eclipse Ni-E, Nikon, Japan) and a confocal microscope (LSM 880, Zeiss, Germen).

#### Anaesthesia induction and sedative administration

To image during anaesthesia, the animals were anaesthetized with isoflurane (1%), and imaging was performed 10min after isoflurane induction. To assess the effects of sedation on NVC, the mice were administered with chlorprothixene (50 μL at 0.33 mg/mL) via i.p. injection 30 min prior to *in vivo* imaging. Experiments typically lasted 1 hour and were followed by recovery.

### Quantification and statistical analysis

#### Image pre-processing

All image processing was carried out using ImageJ (NIH, US) and MATLAB (MathWorks). Image registration was performed using the TurboReg plugin in ImageJ (Thevenaz et al., 1998), to facilitate analysis of the acquired image sequence and correct for any rigid motion artefacts that occurred during *in vivo* imaging.

#### Haemodynamic correction for 1P Ca^2+^ imaging

The recorded GCaMP6 fluorescence was accompanied by artefacts of haemoglobin absorption. These artefacts can be minimised using an empirical correction method. Briefly, as the wavelengths of GCaMP6 fluorescence are close to those of the green LED used in IOS imaging, the haemoglobin absorption of the GCaMP6 fluorescence signal $\left(\Delta \text{F}\left(\text{t}\right)/\text{F}\left(t_{0}\right)\right)$ can be compensated for by the reflected IOS $\left(\text{R}\left(\text{t}\right)/\text{R}\left(t_{0}\right)\right),$ via the following simple division approach (Ma et al., 2016b):

$$\frac{\Delta \text{F}\left(\text{t}\right)}{\text{F}\left(t_{0}\right)}/\frac{\text{R}\left(\text{t}\right)}{\text{R}\left(t_{0}\right)}\approx \frac{\Delta F_{corr}\left(\text{t}\right)}{F_{corr}\left({\text{t}}_{0}\right)}$$

where $\Delta F_{corr}\left(\text{t}\right)/F_{corr}\left({\text{t}}_{0}\right)$ represents the corrected GCaMP6s fluorescence signal.

#### IOS imaging analysis

Tissue absorption is dominated by that of haemoglobin in the visible range (400–700 nm). As HbO_2_ and HbR have the same absorption coefficients at 530 nm, the acquired reflectance ($\text{R}\left(\text{t}\right)/\text{R}\left(t_{0}\right),$ with $t_{0}$ representing the pre-stimulus time point) at this wavelength was related to changes in total haemoglobin concentration $\left(\Delta HbT\;=\;\Delta HbO_{2}+\;\Delta HbR\right),$ as derived from the modified Beer-Lambert law (Hillman, 2007):

(Equation 1) $$\Delta HbT\;=-\frac{1}{\alpha }ln\frac{R\left(t\right)}{R\left(t_{0}\right)}$$

where $\alpha =\frac{1}{2}\left(\varepsilon_{HbO,530}+\varepsilon_{HbR,530}\right)\chi_{530},$ is spatially and temporally invariant, and only depends on the estimated mean path-length of 530 nm light $\left(\chi_{530}\right)$ and the molar extinction coefficient for HbO_2_ and HbR at 530 nm $\left(\varepsilon_{HbT,530}\right).$ Therefore, we used Equation (1) to calculate and analyse the change in CBV (ΔCBV), which is linearly proportional to the change in total haemoglobin (ΔHbT). To reduce the spatial noise in αΔHbT(x,y,t) images, the baseline image $R\left(x,y,t_{0}\right)$ was calculated by applying Gaussian blurring (σ = 1.1 pixels) to the average-intensity projection of pre-stimulus IOS images. For dual-wavelength IOS imaging of the cerebral haemodynamics, the relative changes in HbO_2_ and HbR concentrations were determined using the haemoglobin absorption spectrum ($\varepsilon_{HbT}\left(\lambda \right)$ and $\varepsilon_{HbR}\left(\lambda \right)$) and the Monte Carlo-derived wavelength-dependent path length (χ(λ)) (Ma et al., 2016b).

#### LSC imaging analysis

The speckle contrast was defined as K=σ/μ, where *σ* and *μ* refer to the standard deviation and mean of the speckle intensity acquired with laser (635 nm) illumination, respectively. The speckle contrast was related to CBF by the following equation:

(Equation 2) $$K\propto \sqrt{\frac{\tau_{c}}{T}+\frac{\tau_{c}^{2}}{2T^{2}}\left[e^{-2T/\tau_{c}}-1\right]}$$

where T is frame exposure time of the camera and the inverse correlation time $1/\tau_{c}$ is assumed to be proportional to the speed of the scattering particles (Boas and Dunn, 2010). For small $\tau_{c}$ values ($\tau_{c}\ll T,$ as acquired using our mesoscope), Equation (2) can be simplified by using the following asymptotic approximation (Tom et al., 2008):

(Equation 3) $$\frac{1}{\tau_{c}}\propto \frac{1}{K^{2}}$$

Thus, CBF was represented by the inverse square of speckle contrast. Here, we used the spatial speckle contrast image $K_{s}\left(x,y\right)$ computed in the spatial domain, with $\sigma_{s}$ and $\mu_{s}$ determined in a neighbouring square region of 7 × 7 pixels. The relative change in CBF was calculated as $\Delta \text{CBF }\left(\text{t}\right)/\text{CBF }\left(t_{0}\right)$, where $\text{CBF }\left(t_{0}\right)$ represents the baseline CBF calculated from the average-intensity projection of pre-stimulus CBF images.

#### Neuronal Ca^2+^ transients

For 2P Ca^2+^ imaging, neuron bodies were visually identified from the maximum-intensity-projection images of GCaMP6s fluorescence and manually circled to delineate regions-of-interest (ROIs). The fluorescence traces, represented as $F_{neuron\_measured}\left(t\right),$ were extracted from different ROIs by averaging the corresponding pixels within each ROI. These traces were then used to estimate the fluorescence traces of the neurons, represented as $F_{neuron\_true}\left(t\right),$ after correcting for neuropil contamination using the following formula (Chen et al., 2013; Kerlin et al., 2010):

$$F_{neuron\_true}\left(t\right)=\;F_{neuron\_measured}\left(t\right)-r\times F_{neuropil\_surrounding}\left(t\right)$$

where $F_{neuropil\_surrounding}\left(t\right)$ represents the fluorescence traces of a neuropil within a 20-μm circular region surrounding the neuron body. The contamination ratio *r* is an empirical value that largely depends on the excitation numerical aperture (NA) used for imaging. We used r=0.7, as it served as a good approximation of the degree of contamination; this was confirmed by the decoupled OFF-neuron/neuropil responses after correction (Figure S10). The corrected fluorescence traces $F_{neuron\_true}\left(t\right)$ were then used to calculate the Ca^2+^ transients $\left(\Delta F\left(t\right)/F_{0}\right)$ for individual neurons, where $F_{0}$ is the baseline fluorescence signal averaged over a 1-s period prior to auditory stimulation. Noise filtering was applied to $\Delta F\left(t\right)/F_{0}$ using an exponentially weighted moving average method (Jia et al., 2011). Auditory-evoked responses were measured for each neuron by averaging $\Delta F\left(t\right)/F_{0}$ over different trials (normally 12–24) for each stimulation. For line-scan recording, a two-dimensional space-time image was acquired along the space axis, following a user-defined laser-scanning path. The fluorescence traces F(t) were extracted by averaging the pixels along the scanning path for targeted neurons, and $\Delta F\left(t\right)/F_{0}$ was calculated as for frame imaging.

#### Vessel dilation measurement

PAs labelled by Texas Red dextran were captured via full-frame imaging. The cross-sectional areas of individual arterioles in each frame were computed by thresholding in Radon space, followed by transformation of the thresholded image back to image space (Barrett et al., 2018; Gao and Drew, 2014). The diameter of the PA was determined as:

$$D=2\sqrt{area/\pi }$$

For horizontal vessels, line-scan images acquired by scanning perpendicular to the vessel lumen were used to measure the vessel diameter *D* at full-width at half maximum (FWHM) along the scanning axis. Data were averaged over a 100-ms window to improve the signal-to-noise ratio. Relative changes in vessel diameter were represented as $\Delta D/D_{0},$ where *D*_0_ denotes the pre-stimulus diameter, and low-pass filtered using a Chebyshev filter to remove intrinsic noise (> 1 Hz) caused by the animal’s breathing and heartbeat (Kleinfeld and Mitra, 2014).

#### RBC flow in capillary

Due to the selective labelling of plasma and RBCs, line-scanning along the capillary lumen resulted in a space-time (x-t) image containing dark streaks that represented RBCs. The streak angle θ (relative to the time axis) was used to measure the flow speed of RBCs, as follows:

$$\text{v}=\frac{\Delta x}{\Delta t}\text{tanθ}$$

where Δx and Δt represent the scanning parameters denoting spatial (μm/pixel) and temporal resolution (ms/line), respectively. The streak angle was estimated using a Radon transform method, (Drew et al., 2010) with a 100-ms sliding window and 20-ms time interval between consecutive windows.

#### Cortex-wide image analysis

Following analysis procedures that were similar to those used for mesoscopic imaging, cortex-wide neuronal activity (ΔF(t)/F) and CBV changes (ΔHbT(t)) were obtained from macroscopic optical imaging data with interleaved dual-wavelength illumination. Hemodynamic correction was performed to remove the artefacts of haemoglobin absorption in GCaMP6s fluorescence. To extract neuronal signals from different brain regions, the wide-field ΔF(t)/F data were decomposed into spatial and temporal components using the LocalNMF method and a standard brain atlas as seeding references (Saxena et al., 2020; Oh et al., 2014).

#### Calculation of neurovascular correlation map

For cortex-wide calcium and IOS imaging of neuronal activity (dF/F) and the concurrent vascular dynamics (dHbT), neurovascular correlation maps (NVCMs) were generated by computing the cross correlation between the matrix of dHbT for all pixels over time and neuronal activity dF/F of each temporal component, resulting in a set of maps whose pixel value represents the correlation coefficient, normalised correlation coefficient and lag time. The NVCMs of normalised/un-normalised correlation coefficient can indicate the degree to which the changes in cerebral blood volume (CBV) over the entire dorsal cortex correlate with the neuronal activity of a specific cortical regions, while the NVCM of lag time shows the relative time delay between the neuronal activation and CBV increase.

#### Statistical tests

All data are presented as means ± standard errors of mean (SEM), unless otherwise specified. Statistical analysis were performed using MATLAB (v.2019a) and GraphPad Prism (v.7). Comparison between datasets was conducted using two-tailed unpaired t test (two groups) or two-way ANOVA. In all tests, p < 0.05 is considered statistically significant. All the statistical details were described in the figure legends.

## Data Availability

• All data reported in this paper will be shared by the lead contact upon request. • This paper does not report original code. • Any additional information required to reanalyze the data reported in this paper is available from the lead contact upon request.
