# Supplementary material for: Study of neurovascular coupling by using mesoscopic and microscopic imaging
Source: iScience. 2021 Sep 25;24(10):103176. doi: 10.1016/j.isci.2021.103176 (PMC8511898; doi:10.1016/j.isci.2021.103176)
Supplement: Document S1. Figures S1–S15 [file mmc1.pdf]

iScience, Volume *24*

Supplemental information

Study of neurovascular coupling by using mesoscopic and microscopic imaging

Congping Chen, Zhentao She, Peng Tang, Zhongya Qin, Jufang He, and Jianan Y. Qu

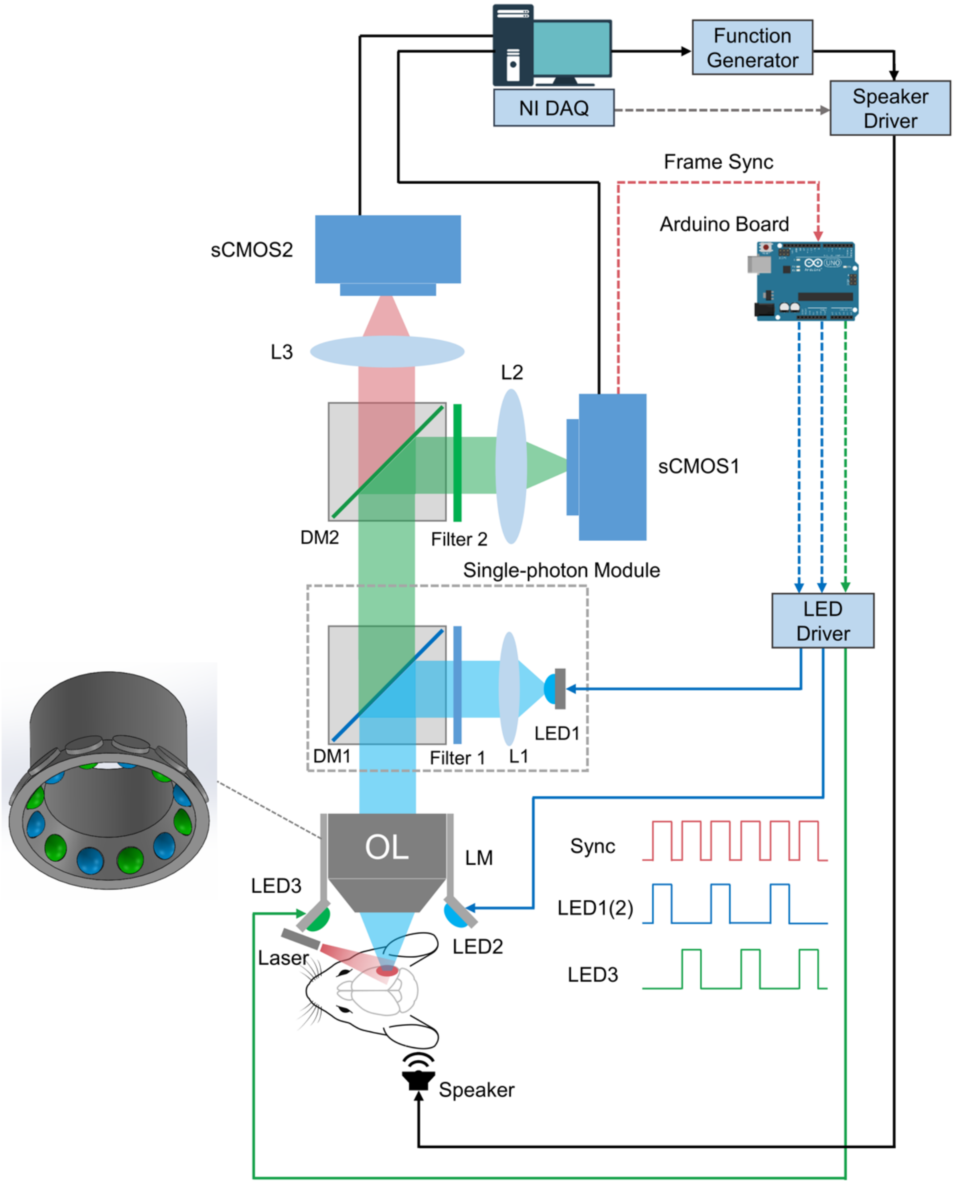

Figure S1. Schematic diagram of simultaneous multi-modal mesoscopic imaging system, Related to STAR Methods. The system integrates three optical imaging modalities, namely single-photon calcium (1P Ca^{2+}) imaging, intrinsic optical signal (IOS) imaging and laser speckle contrast (LSC) imaging. The light source for 1P Ca^{2+} imaging is provided by a 1P module consisting of light-emitting diode (LED)1 (460 nm), dichroic mirror (DM1; T470lpxr) and Filter 1 (band-pass, 460 nm/60 nm). IOS imaging can be performed either in single- (530 nm) or dual-wavelength (530 nm/460 nm) mode by selecting the appropriate LEDs placed in the three dimensionally printed LED mount (LM) for illumination. For simultaneous 1P Ca^{2+} and single-wavelength (530 nm) IOS imaging, the transistor-transistor logic (TTL) signal from the scientific complementary metal-oxide-semiconductor 1 (sCMOS1) camera indicating its light exposure is down-sampled by the Arduino board and used to drive LED1 and LED3 in an interleaved manner. For dual-wavelength IOS imaging, the 1P module can be removed and LED2/LED3 are modulated by the down-sampled TTL signal. The laser speckle images are captured by sCMOS2, with the speckle generated by shining a red laser (635 nm) onto the cortex. For auditory stimulation, white noise or sinusoidal amplitude modulation waves at different frequencies (3–48 kHz) from a synthesised functional generator (DS345, Stanford Research) are amplified and fed into a free-field speaker (MF1, Tucker-Davis Technology) placed 10 cm from the left ear of the mouse.

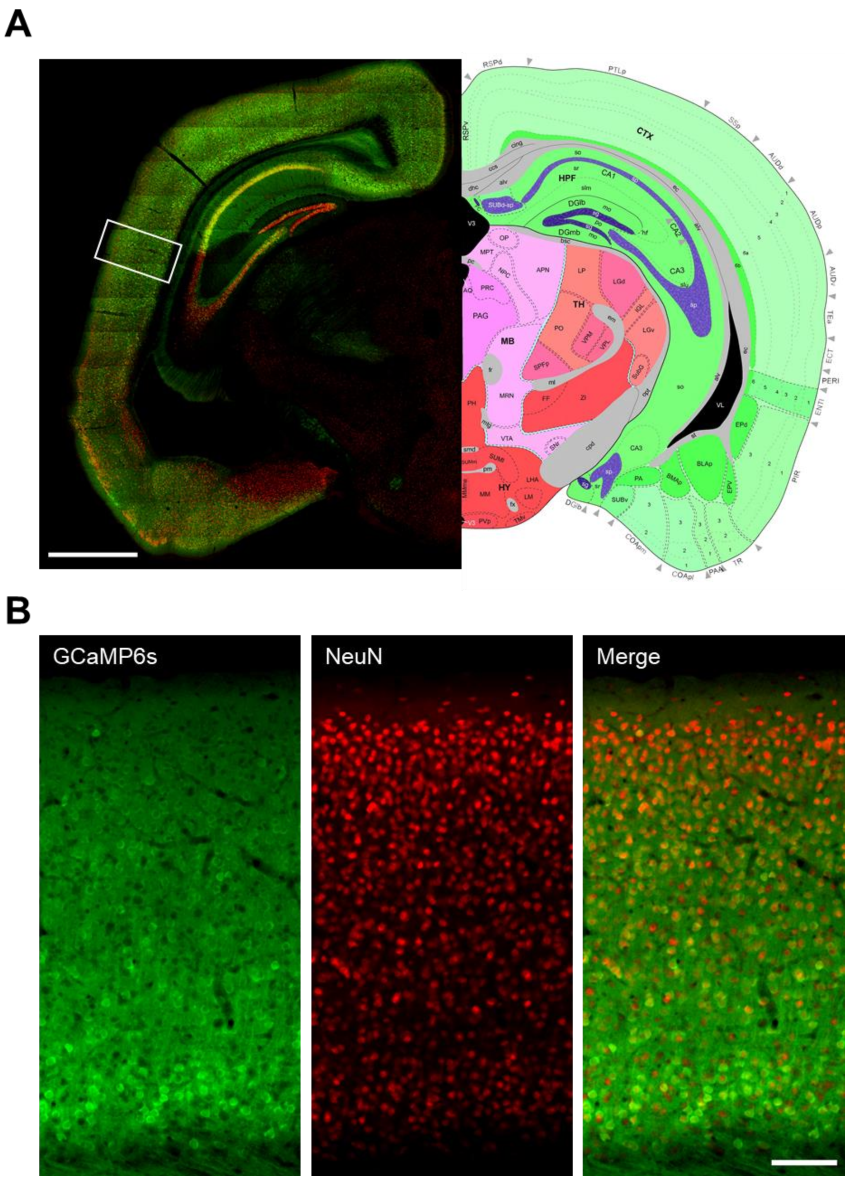

Figure S2. Immunochemistry of transgenic CaMKII-GCaMP6s mice, Related to STAR Methods. (A) Immunostained coronal slice (50 µm thick, ~2.5 mm from caudal to bregma) from an adult transgenic mouse. The corresponding Allen brain atlas and the slice image are placed side by side for comparison. NeuN was labelled by DyLight 405 (emission peak at 420 nm) and is shown in red. GCaMP6s is shown in green. Scale bar: 1 mm. **(B)** High-magnification view of the boxed region (from the auditory cortex) in (A). Scale bar: 100 µm.

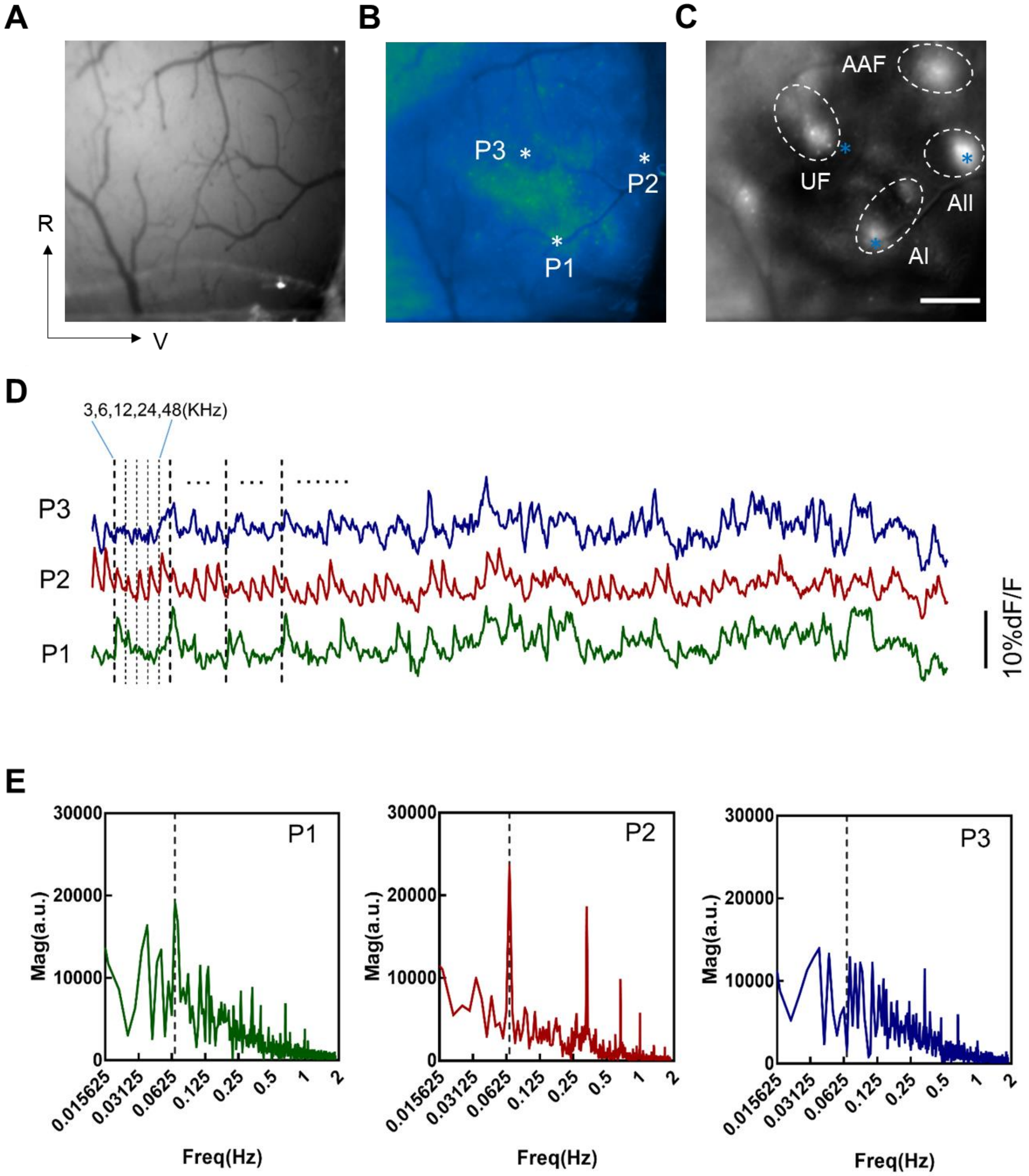

Figure S3. Functional mapping of auditory cortex of CaMPKII-GCaMP6s mice using Fourier imaging, Related to STAR Methods. (A) Reflectance image of auditory cortex with 530-nm illumination, and **(B)** the corresponding wide-field calcium imaging in awake mice. Image was shown as standard-deviation projection of 1800 frames recorded at 7.5 frames/second. Anatomical directions: rostral (R); ventral(V); **(C)** Tonotopic map of the auditory cortex was acquired by applying Fourier transform to the $dF/F\ (x, y, t)$ data and extracting the magnitude component at stimulation frequency. The dashed contours outline the identified auditory fields. AI: primary auditory field; AII: secondary auditory field; AAF: anterior auditory field. UF: ultrasonic field. Scale bar: 0.5mm. **(D)** Calcium transients (dF/F) extracted from three typical pixels (P1,P2,P3) indicated as asterisk in (B). A sequence of single tones with ascending frequencies (3,6,12,24,48 KHz) were presented during calcium imaging at a periodic manner (15s interval), yielding a stimulation frequency of 1/15Hz. **(E)** Magnitude spectra extracted from the three pixels (P1,P2,P3) in (C). The dashed line indicates the stimulation frequency.

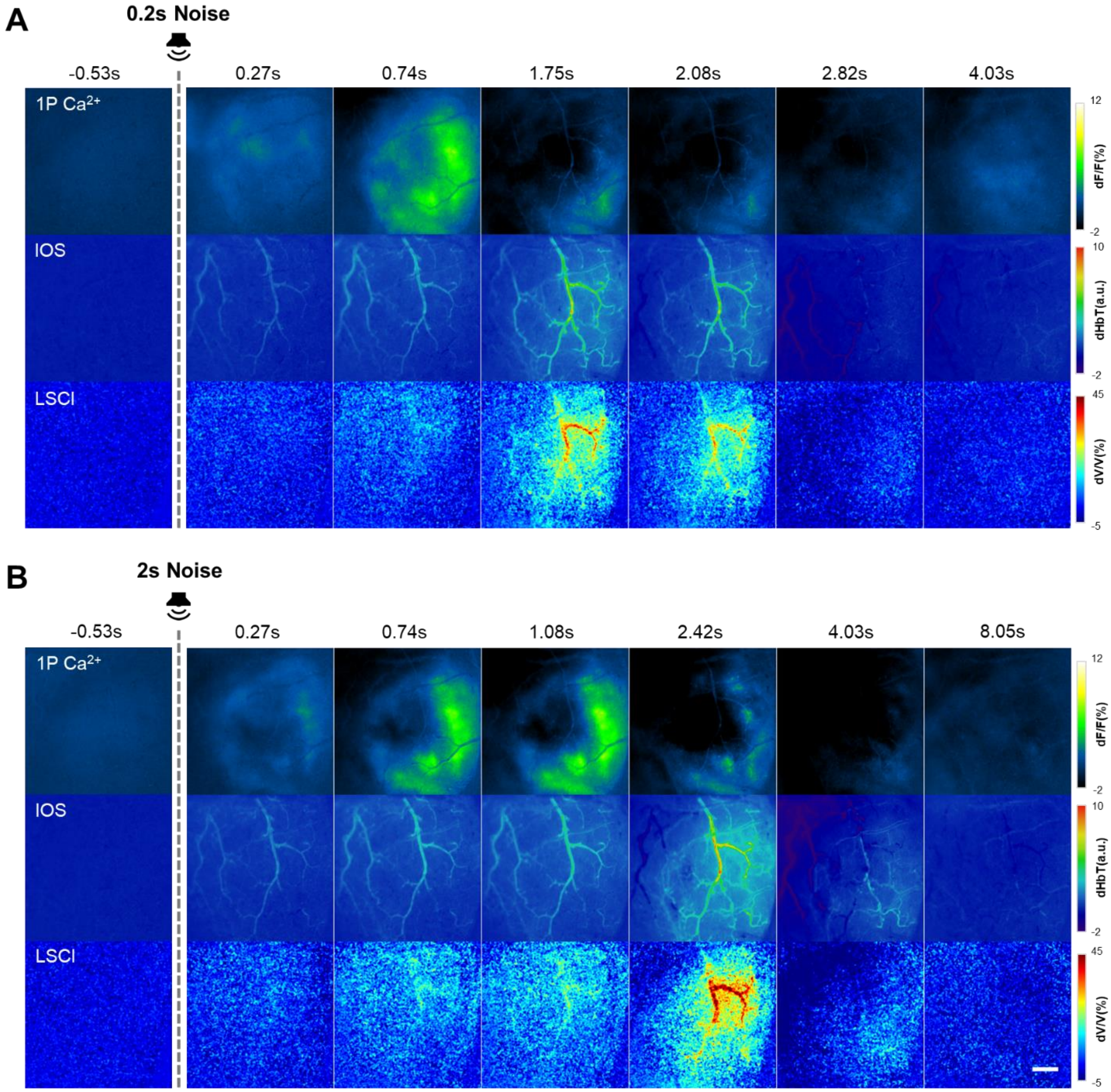

Figure S4. Time course of neuronal and vascular responses in the auditory cortex when subjected to short- and long-duration stimulation, Related to Figure 1. (A-B) Neuronal activities (dF/F), changes in cerebral blood volume (CBV; dHbT) and cerebral blood flow (CBF; dV/V) at different time points with 80-dB auditory stimulation of short- (**A**, 0.2 s) and long-duration (**B**, 2 s). Images were captured at 15 frames/second and averaged over 12–24 trials. Scale bar: 0.5 mm.

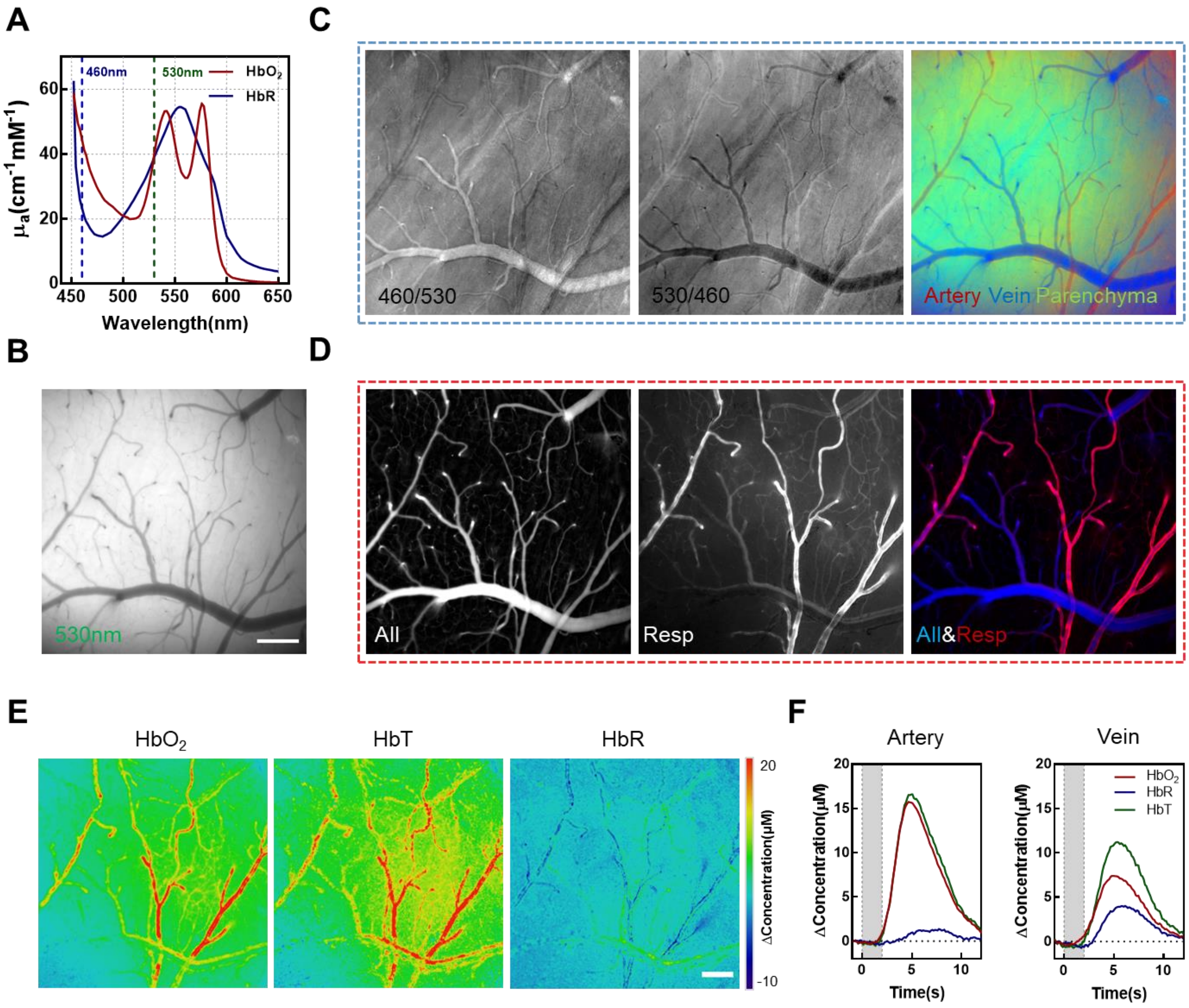

Figure S5. The pial artery and vein show distinct haemodynamic responses to sound stimulation, Related to Figure 1. (A) Absorption spectra of oxyhaemoglobin and deoxyhaemoglobin (HbO_2 and HbR, respectively) in the visible region. Dual-wavelength intrinsic optical signal (IOS) imaging in this study exploited 530-nm (isobestic point) and 460-nm light sources to measure the concentration changes in total haemoglobin (HbT), HbO_2 and HbR. **(B)** Reflectance image of auditory cortex with 530-nm illumination. Scale bar: 0.2 mm. **(C)** Separation of pial artery and vein based on their distinct HbO_2/HbR ratios. Large veins (dominated by HbR) and arteries (dominated by HbO_2) on the cortical surface were identified based on their 460-nm/530-nm (left) and 530-nm/460-nm (middle) ratio images, respectively. The RGB image (right) was created by merging 460-nm/530-nm (blue) and 530-nm/460-nm (red) ratio images with a 530-nm reflectance image (green). **(D)** The pial artery, but not the vein, was the main vascular component showing responses (HbT increase) to external stimuli. Left: inversed reflectance image showing all surface vessels; middle: responsive vessel image acquired by Fourier imaging; right: merge of left and middle images, showing that the responsive vessels consisted exclusively of pial arteries, as confirmed in **(C)**. **(E)** Unmixed concentrations of HbO_2, HbR and HbT. Scale bar: 0.2 mm. **(F)** Time course of averaged concentration changes in HbO_2, HbR and HbT in the artery (left) and vein (right).

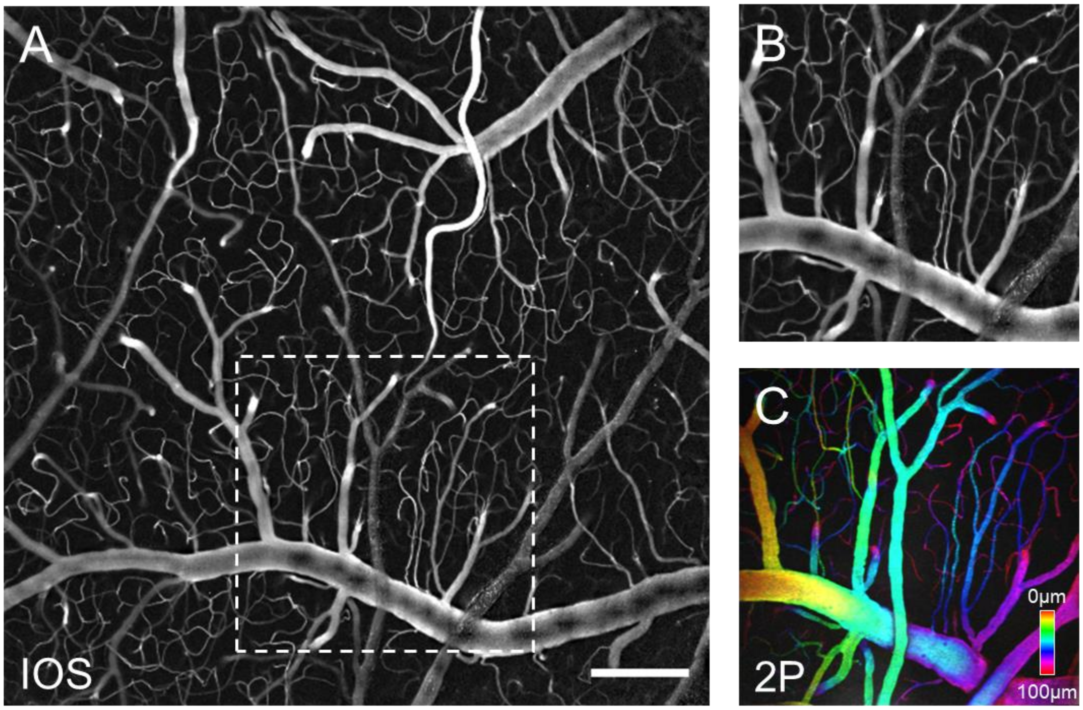

Figure S6. Comparison of vasculature imaging using intrinsic optical signal and two-photon excited fluorescence, Related to Figures 1,2. (A) Label-free image of cortical vessels obtained via dual-wavelength (460-nm and 530-nm) intrinsic optical signal (IOS) imaging. The image is shown as the standard-deviation projection of the 460-nm/530-nm ratio over 900 frames, with the constant background in the brain parenchyma largely reduced. Scale bar: 0.2 mm. **(B)** Magnified image of the dashed rectangular box in **(A)**, and **(C)** the corresponding two-photon fluorescence image of the vessels labelled with Evans Blue (10µg/g; E2129, Sigma-Aldrich). The image is shown as a depth projection from 0–100 µm below the pia. These results show that IOS imaging under our conditions can only resolve the superficial vessels to a depth of 100 µm.

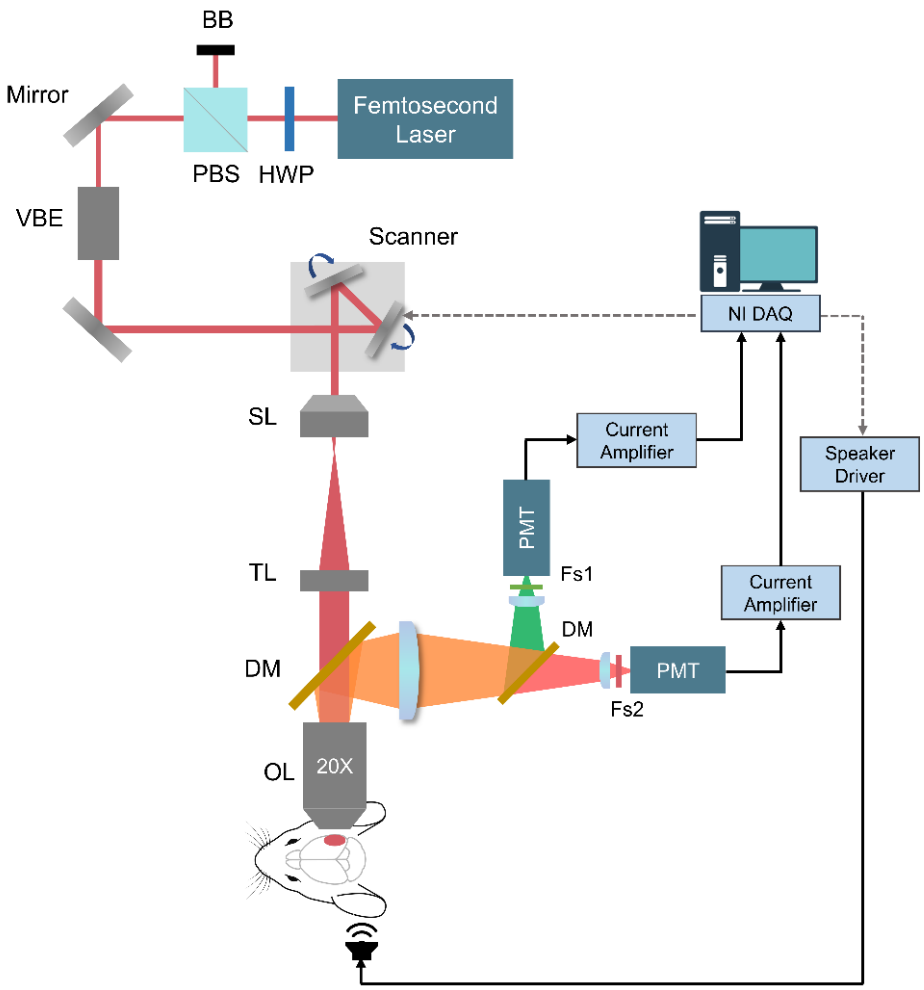

Figure S7. Schematic diagram of the custom-built two-photon microscope system for simultaneous imaging of neuronal activities, arteriolar dilation and blood flow in capillaries, Related to STAR Methods. BB: beam blocker; PBS: polarised beam splitter; HWP: half wave plate; VBE: variable beam expander; SL: scan lens; TL: tube lens; DM: dichroic mirror; OL: objective lens; PMT: photon multiplier tube; Fs: filter set.

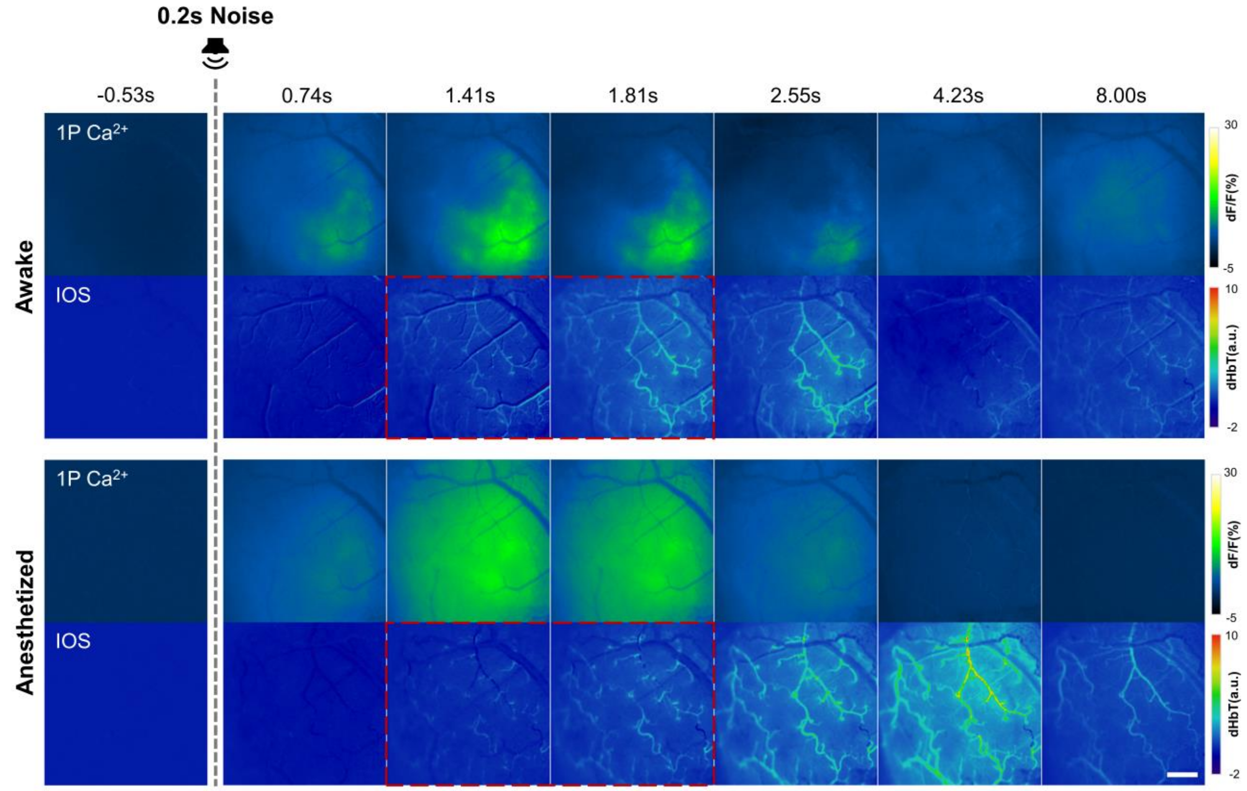

Figure S8. Time course of neuronal and vascular responses in the auditory cortices of awake and anaesthetised mice, Related to Figure 3. Neuronal activities (dF/F) and changes in cerebral blood volume (CBV; dHbT) at different time points upon auditory stimulation are shown. The images were captured at 15 frames/second and averaged over 12–24 trials. For the neuronal response, the areas that were responsive to auditory stimulation were enlarged in anaesthetised mice. The areas marked by a red rectangle show different onset responses for large and small arteries in anaesthetised mice. The dilation of large arteries was more delayed than that of small arteries/arterioles in anaesthetised mice. Scale bar: 0.5 mm.

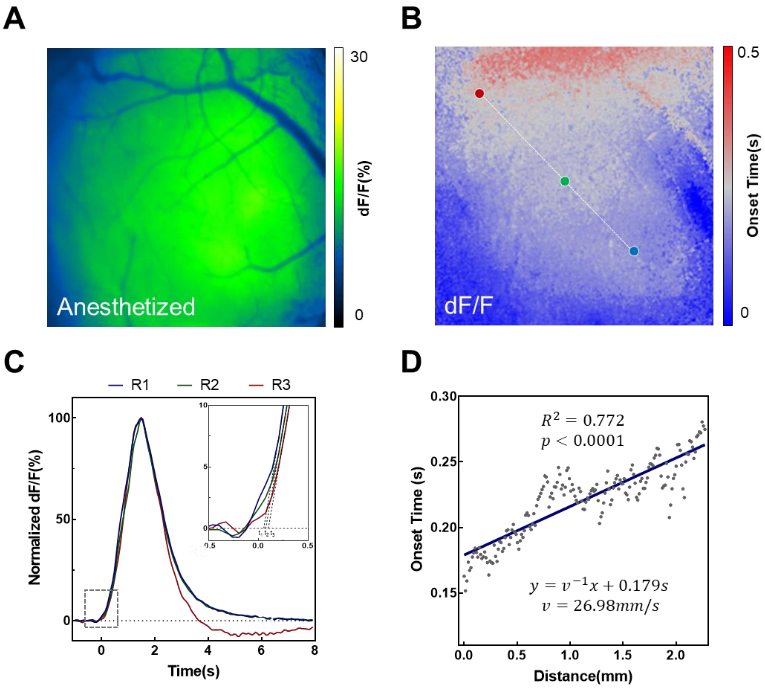

Figure S9. Initiation and propagation of evoked neuronal response under anaesthesia, Related to Figure 3. (A) Amplitude map of neuronal and vascular responses to auditory stimulation (0.2-s duration) in anaesthetised mice and **(B)** The corresponding map of dF/F onset times. **(C)** Normalised dF/F responses from the three dotted regions (R1–3) indicated in (B), with the inset showing the magnified view of the box outlined in the dashed line. Onset time t is defined as the intercept between the linear fitted line of the rising slope (20–80% of peak amplitude) and the pre-stimulus baseline. **(D)** dF/F onset time values selected along the white line indicated in (B). The inverse slope v of the linear regression indicates the propagation speed of the response onset. The estimated speed of onset time propagation along the direction for the mouse is 29.19±7.35 mm/s (mean ± standard deviation, calculated for N = 7 parallel lines).

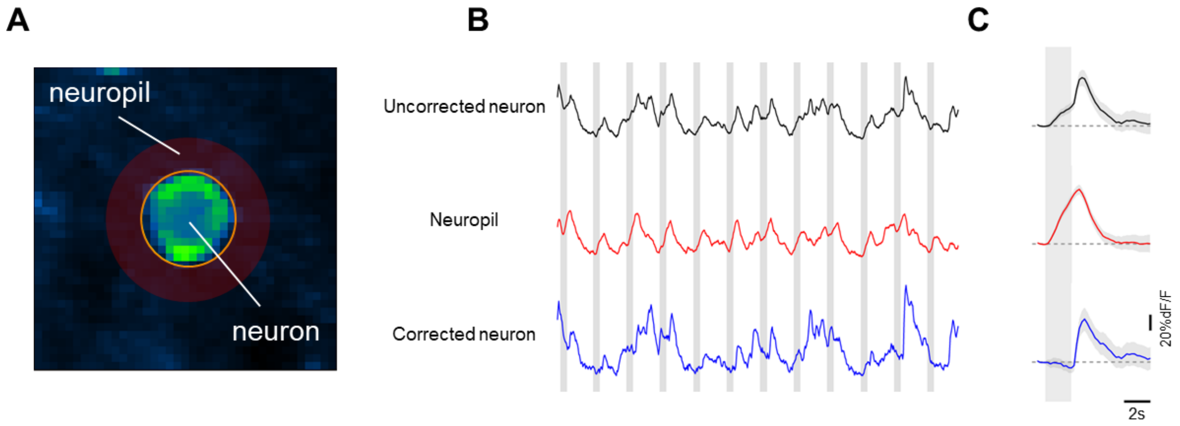

Figure S10. Removal of neuropil contamination is crucial for the accurate interpretation of auditory-evoked neuronal responses, Related to STAR Methods. (A) Neuron bodies were visually identified and manually circled (yellow) to extract the fluorescence trace $F_{neuron\_measured}(t)$. The neuropil trace $F_{neuron\_neuropil}(t)$ was measured from the surrounding circular region. The neuronal fluorescence trace was estimated using the formula $F_{neuron\_true}(t) = F_{neuron\_measured}(t) - r \times F_{neuropil\_surrounding}(t)$, with contamination ratio r = 0.7. **(B)** Ca^{2+} transients of uncorrected neuron, neuropil and corrected neuron. Grey stripes indicate a noise stimulation of 2-s duration. **(C)** Normalised average response of an uncorrected neuron, its surrounding neuropil and the corresponding corrected neuron. The corrected neuron (OFF-neuron) only responds to auditory termination, without any interference from the neighbouring neuropil that shows an ON-response to auditory stimuli.

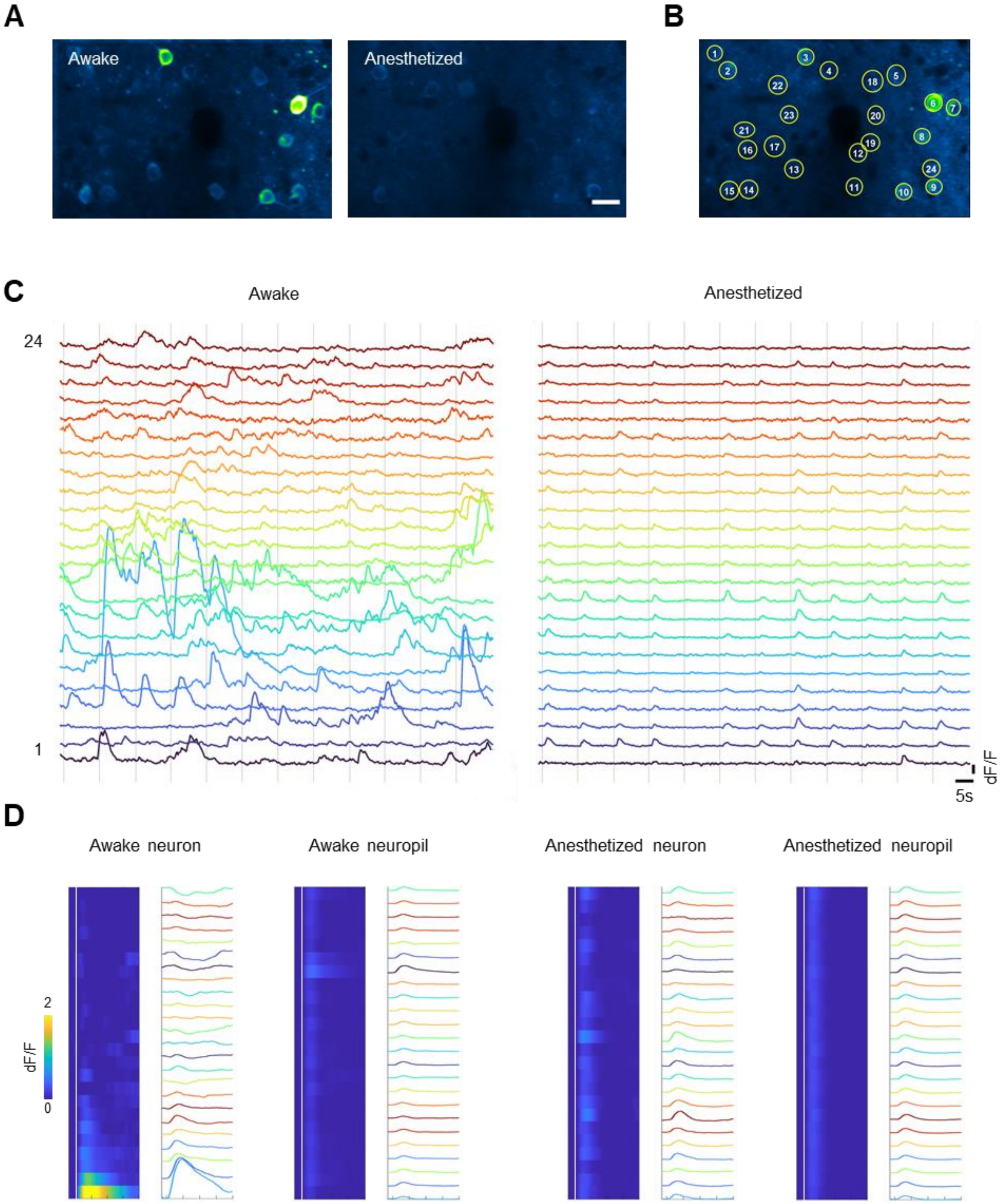

Figure S11. Effects of anaesthesia on the auditory-evoked responses of neurons and neuropil, Related to Figure 3. (A) Calcium (Ca^{2+}) imaging of auditory-evoked neuronal responses at a depth of 220 µm below the pia in the auditory cortex of awake and anaesthetised CaMKII-GCaMP6s mice. The images are shown as standard-deviation projections over 600 frames. Scale bar: 20 µm. **(B)** Fluorescence traces extracted from the same neuron population as indicated by the manually drawn regions of interest and the **(C)** corresponding Ca^{2+} transients for each identified neuron in awake and anaesthetised mice. **(D)** Sorted average responses of neurons and the surrounding neuropil in awake and anaesthetised mice.

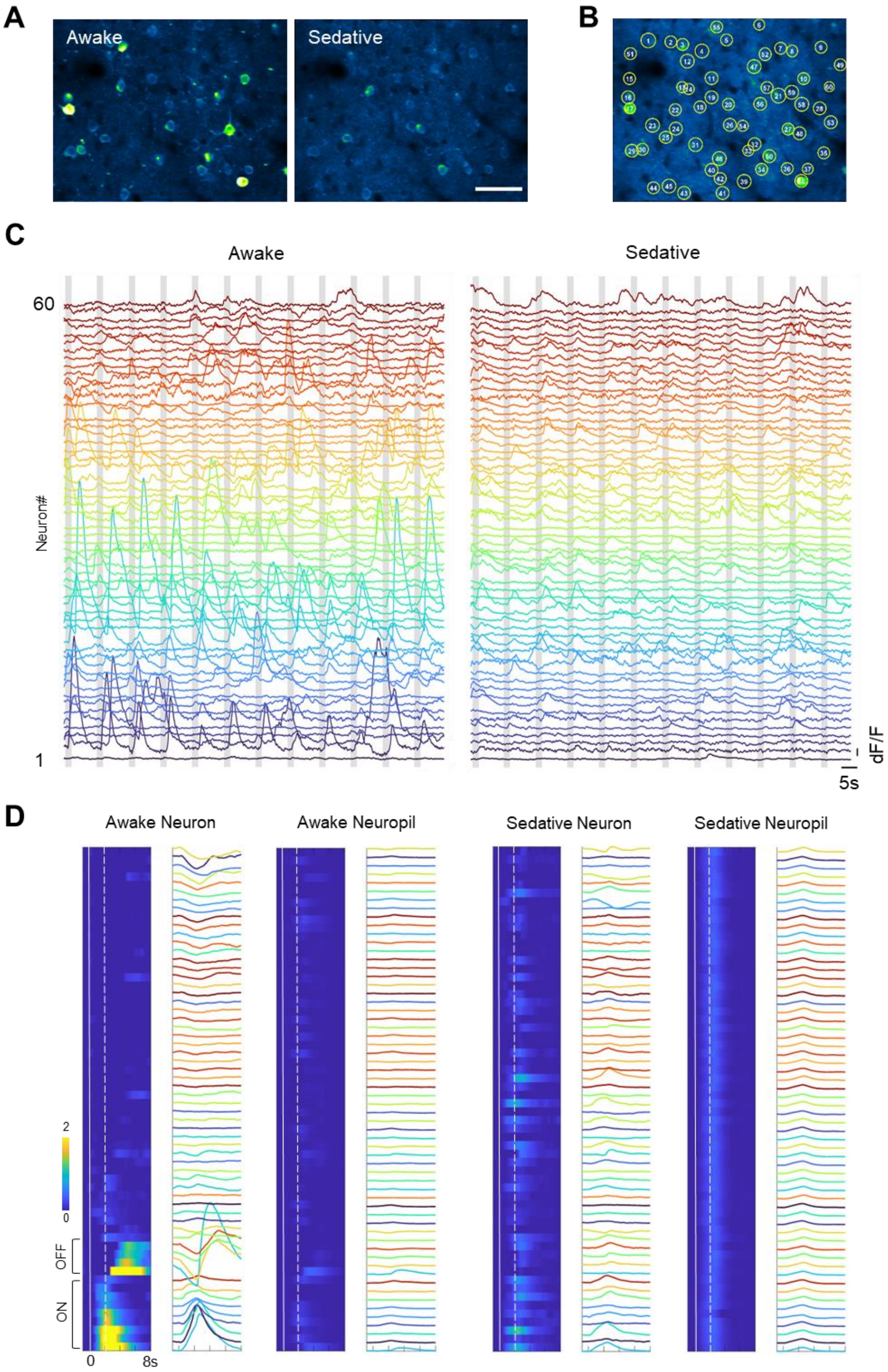

Figure S12. Effect of sedation on the auditory-evoked responses of neurons and neuropil, Related to Figure 4. (A) Calcium (Ca^{2+}) imaging of sound-evoked neuronal responses at a depth of 250 µm below the pia in the auditory cortex of awake and anaesthetised CaMKII-GCaMP6s mice. The images are shown as standard-deviation projections over 600 frames. Scale bar: 50 µm. **(B)** Fluorescence traces extracted from the same neuron population as indicated by the manually drawn regions of interest and the **(C)** corresponding Ca^{2+} transients for each identified neuron in awake and anaesthetised mice. **(D)** Sorted average responses of neurons and the surrounding neuropil in awake and anaesthetised mice. The auditory neurons with ON or OFF responses were identified.

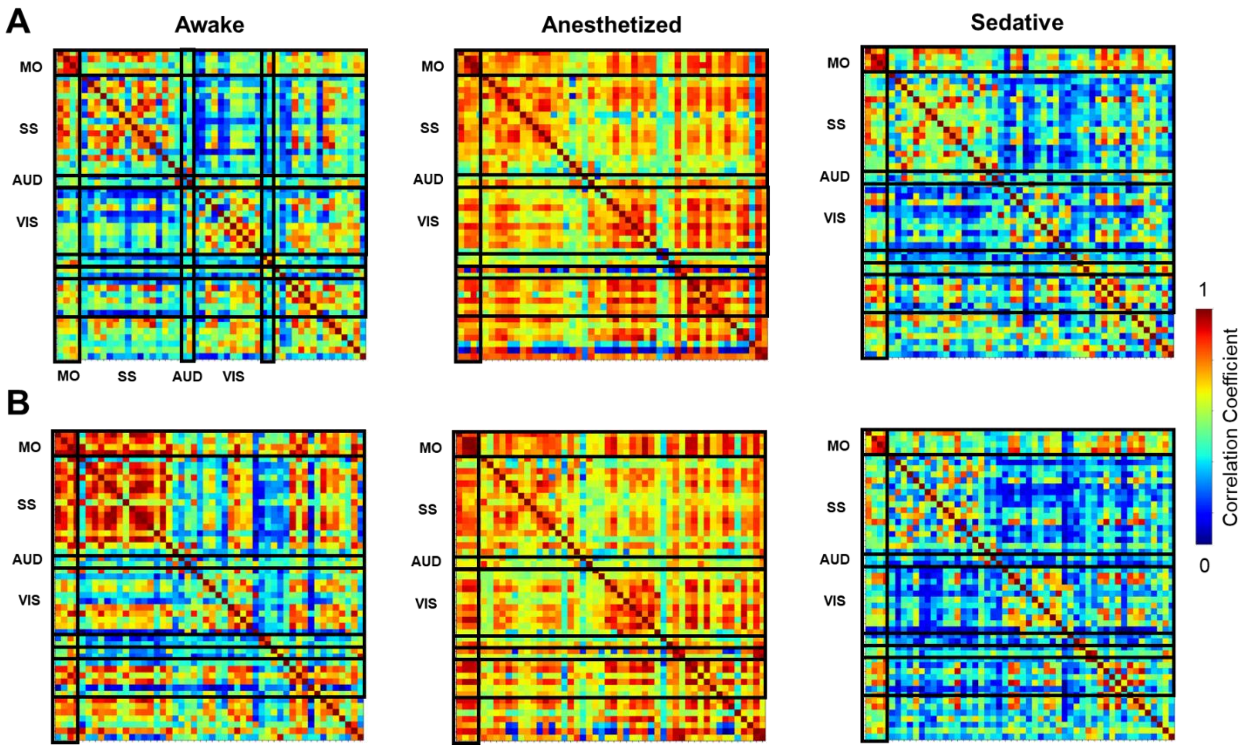

Figure S13. Correlation maps of various cortical regions under different brain-states, Related to Figure 5. (A) Correlation maps of spontaneous activities in the cerebral cortex and **(B)** correlation maps calculated from the auditory-evoked activities in the cerebral cortex. Correlation coefficients between the two cortical regions are calculated from the extracted GCaMP6s calcium transients and displayed in different colours. MO: motor cortex, SS: somatosensory cortex, AUD: auditory cortex, VIS: visual cortex.

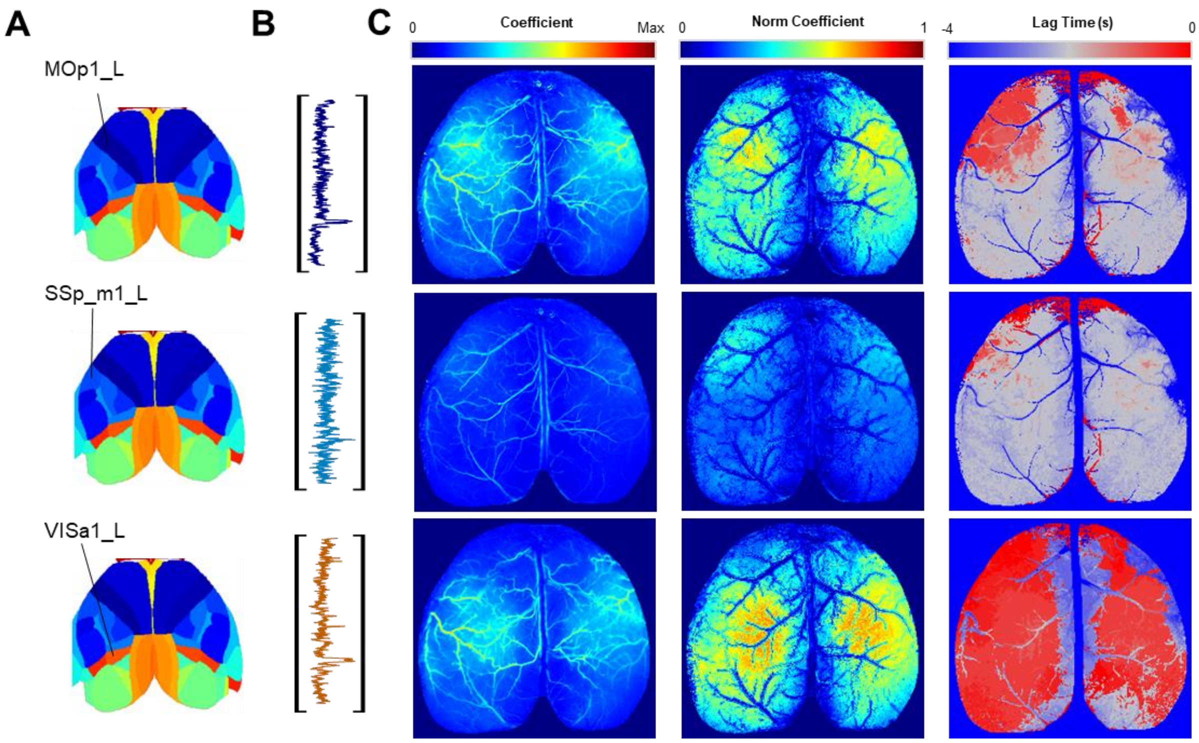

Figure S14. Neurovascular correlation maps calculated from different spatially localised neuronal components, Related to Figure 5. (A) Colour-coded cortical atlas based on anatomical parcellation. The three functional regions (MOp1_L, SSp_m1_L, VISa1_L) are indicated by black lines. **(B)** The corresponding spatial components of calcium (Ca^{2+}) transients were extracted using the localised semi-nonnegative matrix factorisation (LocalNMF) decomposition method. **(Ç)** The corresponding neurovascular correlation maps showing the coefficient (left), normalised coefficient (middle) and lag time (right) of the cross-correlation between the Ca^{2+} transients and cerebral blood volume (CBV; change in total haemoglobin (dHbT)) dynamics.

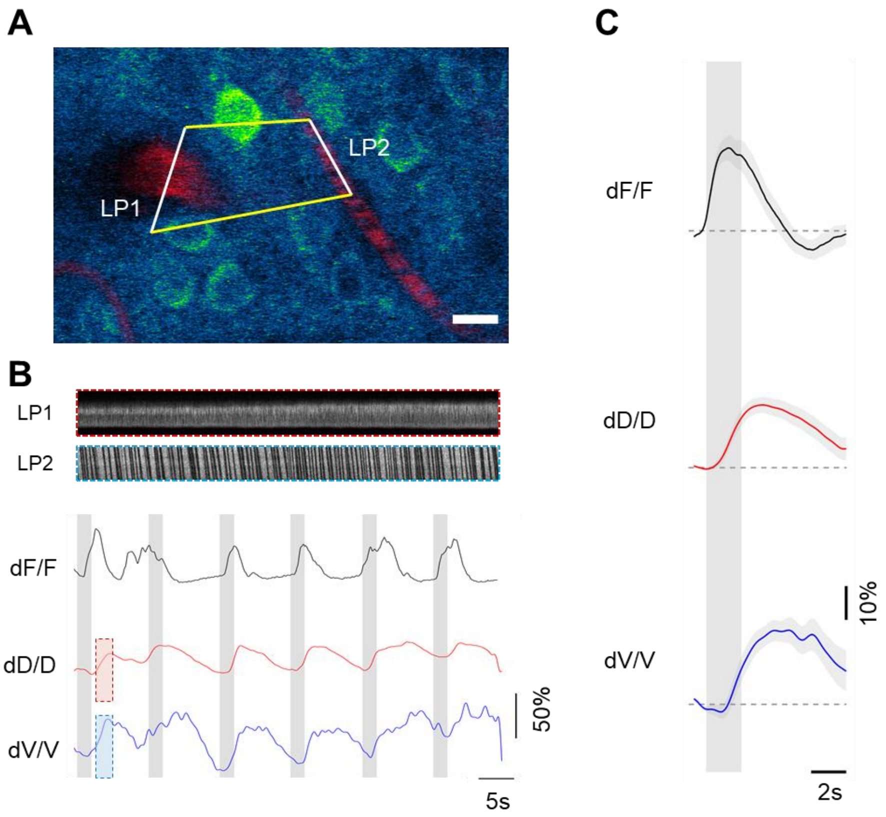

Figure S15. Association between arteriole dilation, red blood cell (RBC) flow in capillaries and neuronal response, Related to STAR Methods. (A) Simultaneous measurement of arteriolar diameter, RBC velocity in capillaries and neuronal Ca^{2+} transients using line-scanning. Line paths (LP1 and LP2) marked in white indicate the scanning period and yellow lines indicate those skipped by laser scanning. Scale bar: 10 µm. **(B)** Dynamics of neuronal activity (dF/F), arteriolar dilation (dD/D) and RBC flow in capillaries (dV/V) evoked by auditory stimulation. The space-time images of LP1 and LP2 correspond to the recording periods indicated by the red and blue rectangles, respectively. **(C)** Average neuronal and vascular responses of auditory stimulation.
